# Combating Ischemia-Reperfusion Injury with Micronutrients and Natural Compounds during Solid Organ Transplantation: Data of Clinical Trials and Lessons of Preclinical Findings

**DOI:** 10.3390/ijms221910675

**Published:** 2021-10-01

**Authors:** Christina Mauerhofer, Lukas Grumet, Peter Schemmer, Bettina Leber, Philipp Stiegler

**Affiliations:** 1 Department of Science and Product Development, pro medico HandelsGmbH, Liebenauer Tangente 6, 8041 Graz, Austria; christina.mauerhofer@promedico.at (C.M.); lukas.grumet@promedico.at (L.G.); 2 Division of Transplant Surgery, Department of Surgery, Medical University, 8036 Graz, Austria; peter.schemmer@medunigraz.at (P.S.); bettina.leber@medunigraz.at (B.L.)

**Keywords:** ischemia-reperfusion injury, reperfusion injury, antioxidants, micronutrients, liver, kidney, heart, transplant, graft, ECD, solid organ transplantation, natural compounds

## Abstract

Although extended donor criteria grafts bear a higher risk of complications such as graft dysfunction, the exceeding demand requires to extent the pool of potential donors. The risk of complications is highly associated with ischemia-reperfusion injury, a condition characterized by high loads of oxidative stress exceeding antioxidative defense mechanisms. The antioxidative properties, along with other beneficial effects like anti-inflammatory, antiapoptotic or antiarrhythmic effects of several micronutrients and natural compounds, have recently emerged increasing research interest resulting in various preclinical and clinical studies. Preclinical studies reported about ameliorated oxidative stress and inflammatory status, resulting in improved graft survival. Although the majority of clinical studies confirmed these results, reporting about improved recovery and superior organ function, others failed to do so. Yet, only a limited number of micronutrients and natural compounds have been investigated in a (large) clinical trial. Despite some ambiguous clinical results and modest clinical data availability, the vast majority of convincing animal and in vitro data, along with low cost and easy availability, encourage the conductance of future clinical trials. These should implement insights gained from animal data.

## 1. Introduction

Solid organ transplantations (SOT) are an important tool in modern medicine as they represent the only curative treatment regime for some end-stage diseases. In 2018, 36,529 SOTs have been performed in the USA according to the Organ Procurement and Transplantation Network data. Currently, there are 113,000 people registered on the waiting list, pointing out the discrepancy between donor organ demand and availability [1]. One attempt to enlarge the pool of possible organ donators is to include extended criteria donors (ECD) who exceed 60 years of age or are between 50 and 59 years of age suffering at least from two of the following conditions: history of hypertension, compromised renal function reflected by terminal serum creatinine > 1.5 mg/dL or cerebrovascular cause of death [2].

Ischemia-reperfusion injury (IRI) is characterized by a stop of blood flow with subsequent restoration of the latter. The initial halt of perfusion induces anoxia, which is paradoxically aggravated by the following reperfusion. This induces different signaling cascades that finally culminate in cell death and a sterile inflammation reaction [3]. During ischemia, mitochondrial respiratory chain is blocked, leading to a sudden ATP drop [4]. The consequential changes lead to a decrease of intracellular pH, as well as an imbalance of cellular ions. This imbalance finally increases the influx of calcium into the cell (reviewed in Reference [5]). The increased calcium content activates hydrolases, e.g., phospholipase A_2_ and proteases, which can induce generation of inflammatory reaction and cause cell damage [6]. Along with intracellular calcium accumulation, sodium accumulation occurs as well, inducing edema that, in turn, induce tissue damage [7]. The increased calcium concentration does not only lead to the activation of various enzymes but also primes mitochondria for mitochondrial transition pore opening, which can finally result in induction of apoptosis [8,9]. However, during conditions of ischemia, the induction of apoptosis is inhibited by the low pH [9,10]. During the ischemic phase, a variety of additional changes are mediated, including the downregulation of intracellular antioxidative defense mechanisms [11,12], as well as the induction of the nuclear factor "kappa-light-chain-enhancer" of activated B-cells (NF-κB) by hypoxia inducible factor 1α (HIF-1α) [13]. 

During reperfusion, the pH quickly restores due to wash-out effects together with an increase of the oxygen levels [5]. Now, different mechanisms occur that do not necessarily have to follow a certain order. Nevertheless, they can fuel each other, further propagating cell and tissue damage [14]. Due to the shift in pH, the imbalance of cellular ions is further exacerbated, yielding an even bigger increase of accumulation of Ca^2+^ ions [5]. This event, together with the “de-inhibition” of mitochondrial transition pore opening due the increase in pH, finally leads to cytochrome C release, mitochondrial swelling and activation of caspases, culminating in the induction of apoptosis [9,15,16]. The final type of cell death is determined by the actual ATP content that remained after ischemia, as apoptosis is an active process that requires ATP. Alternatively, necrotic cell death is initiated [17]. The induced cell damage liberates cellular structures that are considered as damage-associated molecular patterns (DAMP) capable of inducing sterile inflammation by the activation of Toll-like receptors (TLRs) [18]. Subsequently, inflammatory cells are recruited to the site of injury, thereby producing reactive oxygen species (ROS) and further proinflammatory cytokines, which ultimately leads to the onset of a vicious circle [17]. Further cellular damage results from an immediate rise of oxygen concentration after reperfusion that meets previously blunted antioxidative defense mechanisms [5]. Due to mitochondrial lesions, the oxygen cannot be properly reduced, resulting in the generation of ROS [14], in addition to the aforementioned generation of ROS by immune cells. As the compromised antioxidative defense cannot compensate for this high amount of oxidative stress, the damage of various molecules, in addition to the further activation of the central inflammation regulator NF-κB, occur [17,19]. 

Although transplantation and organ preservation techniques are evolving and improving continuously, the IRI-associated initial graft reaction is still a success-limiting factor. This especially applies for organ transplantation of ECDs [1,20]. Recently, micronutrients and natural compounds have emerged increasing interest for supporting various clinical conditions. Especially, reactive oxygen species play a key role in the devastating effects of IRI, so it seems apparent to utilize their antioxidative properties [14]. In addition to their antioxidative properties, specific mechanisms of prevention of IRI have been observed. Surprisingly, a plethora of different micronutrients and natural compounds have been screened for their protective effects already. 

The scope of this review is to provide the reader with a broad overview of currently ongoing research. In this respect, interesting candidates are introduced to give an inspiration for further research on this topic. In order not to overwhelm the reader, we restricted the manuscript to selected micronutrients and natural compounds. Additionally, we solely focused on the three most transplanted organs: heart, liver and kidney, accounting for 99% of all SOTs [1]. To illustrate the full potential of selected compounds, the current research gaps were filled with alternative settings of IRI. This especially applies for animal models, where the protective effects against IRI are not necessarily linked to the setting of SOT to provide valuable information about underlying mechanisms. A tabular summary of the clinical, as well as preclinical, results can be found in Table 1 and Table 2, respectively. Used abbreviations are summarized in Table 3.

## 2. Alpha Lipoic Acid 

Alpha lipoic acid (ALA) is a powerful antioxidant that is registered in several countries for the treatment of diabetic polyneuropathy [21]. It naturally occurs in mitochondria, where it acts as a cofactor for various enzymes that are involved in metabolism [22]. ALA is active in aqueous, as well as in lipid, media. Its antioxidative mechanism is based on the regeneration of antioxidative vitamins C and E, as well as the restoration of endogenous glutathione (GSH) levels. Furthermore, it can directly scavenge ROS and chelate transition metal molecules [14]. In addition to its antioxidant properties, it is known to be anti-inflammatory by the inhibition of NF-κB [22]. 

### 2.1. Kidney

So far, only one preliminary clinical study by Ambrosi et al. [21] has investigated the positive effects of ALA against IRI during simultaneous kidney–pancreas transplantation. Pretreatment with ALA could reduce proinflammatory cytokines and upregulate endogenous antioxidants, as reduced serum levels of interleukin 8 (IL-8) and interleukin 6 (IL-6), as well as the upregulation of hemoxygenase 1 (HO-1), have been observed, respectively. In addition to improved inflammatory markers, protective effects on grafts could be observed as well. A pretreatment with ALA reduced the extent of kidney dysfunction and clinical pancreatitis. However, despite the observed short-term benefits, no effect on the 3-month survival has been reported. Interestingly, the authors observed more pronounced effects if donors and recipients were treated rather than only recipients. For this reason, they suggested to supplement the organ preservation solution with ALA, as this is a less complicated way to treat the graft prior to transplantation. Preclinical studies further support the kidney-preserving [23,24,25] and antioxidative effects [23,26]. Some additional effects that were proposed for ALA in animal models are its attenuation of IRI-induced decreased expression of aquaporins and sodium transporters [24], protection of renal endothelium [24] as well as a decrease of creatine and urea levels [25].

### 2.2. Liver

Only a limited number of clinical trials investigating the beneficial effects of ALA in the setting of liver IRI have been conducted. Casciato et al. [27] reported about a reduced frequency of postperfusion syndrome in liver transplantation patients after pretreatment of grafts and recipients. Furthermore, a trend towards a reduced number of rejections has been observed. On the cellular level, ambiguous results on the inhibition of apoptosis, as well as anti-inflammatory effects, have been observed. However, a subgroup analysis including only ECD grafts revealed beneficial effects on the bilirubin levels. Protective effects against IRI on hepatic tissue have also been observed in a study on patients undergoing liver resection. In order to minimize blood loss and improve recovery of the patients, the portal triad has been clamped via the pringle maneuver [28]. Albeit minimizing blood loss, this approach bears the risk of IRI. Pretreatment with ALA preserved liver function, as indicated by reduced serum levels of alanine aminotransferase (ALT) and aspartate amino transferase (AST), as well as decreased levels of apoptotic makers. Interestingly, the beneficial effects on graft health were associated with the preservation of tissue ATP content. The authors speculated about the antioxidative properties of ALA as the underlying mechanism. By regulating the redox balance, ALA would be able to preserve crucial ATP disulfide bridges that are prone to oxidation in the course of IRI. 

In animal studies, the preservation of tissue integrity as reflected by reduced levels of lactate dehydrogenase (LDH) [29], ALT [30,31] or AST [30], in addition to an increased ATP content [29], has been observed. Preservation of the ATP content has been observed in the clinical study [28] as well). In addition to direct anti-apoptotic effects, anti-inflammatory [29,30,31,32] and antioxidative effects [30,31] have been described. The preserving effects on grafts finally translated into increased survival of treated animals [33,34]. 

### 2.3. Heart and Vessels

To the best of our knowledge, no clinical studies on IRI in the course of heart transplantation have been conducted. However, a Turkish study [35] performed in 2013 observed attenuated levels of proinflammatory cytokines IL-6 and IL-8 after the addition of ALA into the priming solution for cardiopulmonary bypass. Additionally, C3 and C4, as well as c-reactive proteins (CRP) that have been raised during reperfusion of the graft, were reduced after pretreatment with ALA. 

Contrary to human clinical studies, a plethora of animal preclinical studies reporting aboutbeneficial effects of ALA against IRI exists. The beneficial effects comprised enhanced graft survival [36] as reflected by reduced LDH and creatine kinase (CK) release [37,38], as well as decreased infarct size [37] and improvement of oxidative stress [39], as well as preserved cardiac function [40]. The latter one is especially associated with a decreased number of arrhythmias [41] and improved coronary flow, as well as increased peak arterial pressure [40]. ALA has been shown to induce aldehyde dehydrogenase (ALDH_2_). This protein is known specifically for the detoxification of aldehydes [39]. Moreover, anti-inflammatory mechanisms by the inhibition of NF-κB and extracellular-signal regulated kinase (ERK) have been observed [42]. However, some of these results remain inconclusive, as other studies could not find preserved cardiac function [43,44], attenuated oxidative stress [43] or effects on injury or inflammation [45]. 

### 2.4. Concluding Remarks

Currently, only a limited number of clinical studies utilizing ALA in the context of IRI in SOT exist. This is especially true for the heart, where no study on heart transplantation has been conducted. However, the effects of ALA on IRI have been demonstrated on patients undergoing coronary artery bypass graft (CABG). Interestingly, superior protection by ALA has been observed when donors, as well as recipients, were treated at least in the setting of kidney and liver transplantation. This interesting finding should be kept in mind in general for IRI prevention regarding future trials, although it has to be proven if it applies for other micronutrients/natural compounds as well. Contrary to human clinical studies, vigorous research activities on animal models regarding the protective effects of ALA in the context of IRI can be observed. In animal models, general systemic effects like protection of the endothelium have been observed, in addition to specific effects on the affected organ, including the attenuation of decreased aquaporin expression in the kidneys and antiarrhythmic effects in the heart. Yet, some preclinical studies investigating the effect of ALA on the heart did not find beneficial effects or even negative effects at very high doses [46]. For this reason, future clinical studies should emphasize determining the proper dosage range.

To sum up, the broad range of effects observed in primal clinical studies, as well as a variety of preclinical studies, warrants the conductance of future large clinical trials in order to elucidate the full potential of ALA. 

## 3. Astaxanthin

Astaxanthin is a fat-soluble carotenoid possessing antioxidative effects. Due to its hydrophobicity and structure, the antioxidative functions of astaxanthin are sometimes compared to vitamin E [47,48], as both compounds appear to localize within membranes and help to attenuate membrane damage. Moreover, the literature suggests that astaxanthin effectively suppresses lipid peroxidation [49,50]. Astaxanthin is usually found in microalgae from which it can be produced easily and inexpensively.

### 3.1. Kidney

In 2008, an Australian group initiated a large randomized controlled trial (RCT) to assess the effect of astaxanthin on arterial stiffness, oxidative stress and inflammation in renal transplant patients. Titled the XANTHIN trial, the study’s results were published 8 years later in 2016. The RCT, involving 61 patients receiving either 12-mg astaxanthin or a placebo orally for 1 year after renal transplantation, showed no effects on the mentioned parameters. While this is an important finding for transplant surgeons aiming to work with astaxanthin, it shall be noted that the study did not specifically address the effects on IRI prevention [51].

Two preclinical studies addressed the effects of astaxanthin specifically against renal IRI in vitro [52] and in animal models [52,53]. In vitro, astaxanthin protected human tubular epithelial cells against H_2_O_2_-induced death. In the animal models, pre-treatment with astaxanthin significantly improved markers of oxidative stress [52,53], cell death and inflammation [53]. Mice receiving astaxanthin also showed reduced levels of circulating urea nitrogen and creatinine, as well as improved histopathological scores. The authors concluded that astaxanthin could be a potential, safe and effective candidate for kidney transplantation in preventing acute injury caused by IR [52].

### 3.2. Liver

Our literature search did not reveal any clinical studies for astaxanthin in the prevention of hepatic IRI. Studies in animal models with special references to the liver are rare; yet, we found three studies addressing the topic. In these animal models, astaxanthin promoted a significantly reduced histopathological scoring [54], reduced activity of prooxidative enzymes [54] in addition to reduced levels of markers indicative of oxidative stress [54,55]. Additionally, tissue integrity was maintained by inhibition of apoptosis [55,56] as well as necrosis [55]. Furthermore, inhibitory effects on autophagy [56] and inflammatory pathways [55] have been observed.

A larger number of animal studies addressing the prevention of IRI through astaxanthin in other solid organs were summarized in the excellent review by the authors of Reference [57] from 2018, which we recommend consulting in order to gain a broader understanding of astaxanthin in IRI in general. 

### 3.3. Heart and Vessels

No clinical studies addressed whether astaxanthin could be used in terms of cardiac IRI prevention. A synthetic form of dietary astaxanthin (disodium disuccinate astaxanthin) has been reported to reduce inflammatory activation of the complement system and myocardial infarct size in a rabbit model of ischemia/reperfusion [58]. 

These results were confirmed in a murine study showing that astaxanthin significantly reduced myocardial infarct size. As underlying mechanism, preservation of mitochondrial integrity reflected by mitochondrial ROS, membrane potential changes as well as swelling has been mentioned. Moreover, the authors observed beneficial effects on IRI-induced increase in plasma and cardiac levels of oxidative stress [59]. Similar effects have been observed in an in vitro study, where administration of astaxanthin protected myocardial cells from hypoxia/reoxygenation injury [60].

### 3.4. Concluding Remarks

In terms of the protective effects of Astaxanthin against IRI in the context of SOT, the clinical trial situation is very modest. No clinical studies have been conducted so far either for kidneys, liver or heart. However, one clinical study addressing kidney transplantation observed the beneficial effects of Astaxanthin administration on the systematic parameters like arterial stiffness, oxidative stress and inflammation. Compared to clinical studies, a variety of preclinical studies exists observing the anti-inflammatory, antiapoptotic and antioxidative properties in all the organs described. 

These promising clinical and preclinical results, in addition to their cost-effectiveness and easy availability, warrant future clinical trials, although this compound has not been investigated as extensively as others.

## 4. Coenzyme Q10

Coenzyme Q10 (CoQ10) is a vitaminoid that occurs ubiquitously in virtually every cell in the body. Its central function is the gradual transfer of electrons and protons to oxygen with the simultaneous production of ATP as a biochemical energy equivalent within the respiratory chain in the mitochondria. In the context of IRI prevention, however, the antioxidant properties [61] of CoQ10 seem to be of particular importance, as the scavenging of ROS remains one of the key challenges in IRI

### 4.1. Kidney

We were unable to find published clinical trials dealing with renal IRI prevention using CoQ10 within or outside the context of transplantation. 

In a rat model, CoQ10 was shown to enhance the rate and extent of ATP resynthesis after warm ischemia. The effects were accompanied by an increased survival rate [62]. Further rat studies also documented improved histopathological scores, improvement of inflammatory markers, decreased levels of oxidative stress as well as improved kidney function due to administration of CoQ10 [63,64,65]. Interestingly, it was postulated that a property apart from the anti-inflammatory, antioxidant, antiapoptotic and antiperoxidative characteristics of CoQ10 could be responsible for the documented reno-protective effects [64]. 

### 4.2. Liver

No clinical studies on the effects of CoQ10 on IRI during liver transplantation have been performed so far. In rats: the intravenous administration of CoQ10 to donor rats and/or recipient rats resulted in 1-week survival rates of 45.5% and 50%, respectively, while rats receiving the placebo did not survive for longer than 2 days. In addition, long-term-surviving rats with transplanted IR-damaged livers pre-treated with CoQ10 showed improved liver biomarkers in serum. The observed parameters reached almost the level of fresh-liver-transplanted control rats [66].

IRI in general could be ameliorated as demonstrated in various animal studies. Amongst others, apoptosis, oxidative stress, inflammation, serum biomarkers, and associated histopathological findings were predominantly improved [67,68,69]. Two further studies investigating the effects of CoQ10 on endogenous homologs (i.e., CoQ9, which is rat’s native ubiquinone) found that administration of CoQ10 protected the respective endogenous homolog levels which ultimately resulted in increased antioxidant power of livers. [70,71]. A study monitoring the survival of rats subjected to hepatic IRI and CoQ10 treatment found that CoQ10 administration increased the survival rate of rats undergoing IR from 0 % to 60 %. While this is a particularly promising observation, it should be noted that we were only able to access the abstract of this respective study [72].

### 4.3. Heart and Vessels

Most of the CoQ10 related studies we found in our organ-specific research concerned the heart. This can probably be explained by the fact that people suffering from heart diseases show a depletion of CoQ10 [73], emphasizing its importance in heart physiology (see, also, Reference [74]). A particularly interesting aspect of CoQ10 in relation to cardiovascular health is that CoQ10 in and of itself has the potential to provide preventive support in heart failure, a fact that eventually makes the need for heart transplants (and, thus, fighting IRI) obsolete in the first place. Berman et al. showed that the preoperative administration of CoQ10 to heart transplant candidates leads to significant improvements in the functional status, clinical symptoms and quality of life [75]. Doctor Sinatra, who operates at the preventive Cardiology Department in Manchester, Connecticut, USA, reported (in a letter, not a study) that two patients managed to get off transplant lists as a result of CoQ10 intake [76].

Another clinical study looked into the effect of preoperative oral CoQ10 on patients undergoing elective cardiac surgery. The patients receiving CoQ10 showed superior mitochondrial respiration, as well as reduced mitochondrial MDA content, compared to patients treated with the placebo. Pectinate trabeculae isolated from patients receiving CoQ10 exhibited a greater recovery of developed force compared with those of the patients receiving the placebo [77]. 

Rosenfeldt et al. excised human atrial trabeculae from older (>70 years of age) and younger (<70 years) patients and subjected them to 30 minutes of ischemia. The researchers then measured the effect of CoQ10 pretreatment on the contractile function recovery, which was significantly different between older and younger individuals (favoring younger individuals). The pretreatment of CoQ10 attenuated the ischemia-induced decline of the contractile force in trabeculae from older patients [78], supporting the finding from Reference [77]. 

Two trials conducted in the 1990s by Karlsson et al. assessed the cardiac and plasma CoQ10 levels in patients subjected to heart transplantation. Both publications reported that postoperative symptoms of graft rejection have been associated with reductions in the CoQ10 levels, particularly in plasma [79,80].

Apart from the trials described above, we found no clinical studies dealing with ischemia-reperfusion injury in terms of cardiac transplant surgeries; yet, there is data on CoQ10 in terms of bypass surgery, which frequently involves periods of ischemia.

A clinical trial from 1994 by Chello et al. investigated the effect of CoQ10 on IRI in coronary artery bypass surgery. The intervention group, which received 150-mg CoQ10 per day 7 days before the surgery, showed significantly reduced levels of conjugated dienes, creatine kinase and MDA after reperfusion had taken place. Furthermore, significantly less incidences of ventricular arrhythmias were observed in the CoQ10 group as compared to the control group [81].

In contrast, another clinical study also investigating the effects of CoQ10 on IRI in coronary artery bypass surgery by applying an equal duration of intake and dosage was unable to observe any significant effects in the context of infarct biomarkers CK-MB and troponin. The randomized clinical trial also failed to demonstrate effects on the postoperative outcomes, such as the duration of intensive care unit (ICU) or hospital stays [82].

In return, a trial investigating the effect of oral CoQ10 supplementation 7–10 days prior elective cardiopulmonary bypass found that CoQ10 therapy significantly improved reperfusion-related arrhythmias, total inotropic requirement, mediastinal drainage, blood product requirement and the hospital stays as compared to patients treated with the placebo [83].

A retrospective analysis of 78 patients undergoing coronary artery bypass grafting pretreated with CoQ10 revealed the effectiveness in preventing left ventricular depression in early reperfusion and in minimizing myocardial cellular injury during bypass grafting followed by reperfusion [84].

Studies on the heart also dominate in the animal models: Whitman et al. isolated hearts from rats pretreated intramuscularly and intraperitoneally with either CoQ10 or the vehicle 24 and 2 h prior to ischemia reperfusion. CoQ10 pretreatment appeared to improve the myocardial function through an increase of aerobic energy production. As such, CoQ10 was able to preserve/delay the hearts’ aerobic efficiency during the precarious reperfusion process [85]. In another rat study, myocardial function could also be preserved via acute administration of CoQ10 before reperfusion [86].

A Russian study of which we were only able to access the English abstract reported cardioprotective effects of CoQ10 injected intravenously into rats 30 min before coronary artery occlusion [87].

CoQ10 was also found to be effective in a porcine open-chest model where it significantly reduced cardiac stunning time [88]. Literature search also revealed protection from IRI through CoQ10 in a rodent model, as Verma et al. infused liposomal CoQ10 into hearts of rabbits through the left atrium. CoQ10 treatment significantly reduced the total area of myocardial tissue being at risk of severe damage by 30% as compared to rabbits receiving vehicle, and by 40% as compared to rabbits receiving krebs-henseleit buffer [89].

Finally, an in vitro study investigating respiratory functions of isolated cardiac mitochondria found CoQ10 to preserve the respective functions through improvement of FADH-dependent oxidation during reperfusion [90].

### 4.4. Concluding Remarks

CoQ10 is especially known for its crucial role in maintaining heart health, as reflected by a CoQ10 deficiency in a variety of heart diseases. However, the compound has only been tested before the actual heart transplantation so far, although a link between graft rejection and CoQ10 levels has been observed. The potential of this compound on IR-induced heart injuries has been proven in studies investigating its protective effects on IRI during CABG. Contrary to studies addressing heart health, no clinical studies on the protective effect of CoQ10 on kidney or liver have been conducted so far. Contrary, a variety of preclinical studies showed beneficial effects on all three organs reported. Interestingly, the impact on the energy metabolism was especially highlighted, reflected by preserved mitochondrial function and increased ATP resynthesis. These effects are less surprising considering CoQ10’s role in the electron transport chain. 

So far, CoQ10 has been appreciated for its crucial role in maintaining heart health. However, preliminary clinical studies, as well as a variety of preclinical studies, point towards its high potential in combating the negative consequences of IRI and, thus, justify future clinical trials.

## 5. Curcumin and Quercetin

Quercetin was originally discovered in 1936, where it was named “vitamin P”, because it was shown to cure a bleeding disorder and stop the “permeability”. Quercetin belongs to the family of flavonoids and is ubiquitously present in our diet. It can be found in all fruits and vegetables, as well as in seeds, tea, nuts and red wine. Although it is considered the most potent scavenger of ROS within the polyphenol family [91], due to its chemical nature, it is hydrophobic and possess poor bioavailability. For this reason, many technical attempts have been conducted to improve its bioavailability. However, quercetin is highly appreciated for its biological properties that include anti-inflammatory, antiplatelet, antioxidative, antihistaminic, anticarcinogenic, antidiabetic, antibacterial, neuroprotective and immunomodulating ones (reviewed in Reference [92]). 

Curcumin is one of the main components of *Curcuma longa*, a root that belongs to the ginger family and is endemic in tropical and subtropical regions, including India, China and Southeast Asia. Besides curcumin, it also contains carbohydrates, proteins, fat, fibers and essential oils. Curcumin is especially known for its antioxidative potential. It is either able to directly scavenge various forms of ROS or to induce the expression of endogenous cytoprotective proteins like SOD, glutathione peroxidase (GPx), HO-1 or catalase (CAT) [93]. Besides its antioxidative effects, it can also mediate anti-inflammatory effects by inhibiting the central inflammation regulator NF-κB [94].

### 5.1. Kidney

The protective effects of quercetin and curcumin have been investigated in two clinical studies. Shoskes et al. performed an open-label pilot study on 15 renal transplant patients [94]. It should be mentioned, that only six of them received the graft immediately before the application of polyphenols and presented with delayed-graft function. The treatment with Oxy-Q, which contained curcumin among other natural compounds, improved the graft function, as urine output and creatine clearance were improved. Moreover, iso-prostaglandins as markers of oxidative stress decreased. A few years later, a follow-up study by the same group was published. The randomized, double-blinded, placebo-controlled study included 43 dialysis patients scheduled for cadaveric kidney transplantation. They received a slightly modified formulation of Oxy-Q, starting within the first 24 hours of transplantation for one month. In the treatment group, a dose-dependent early graft function was observed to a significant degree. Improvements in creatine clearance and smaller numbers of acute rejection events within 6 months further proved the beneficial effect on graft function. On the cellular level, polyphenol-treatment increased the expression of the endogenous antioxidant HO-1 but did not influence total antioxidant capacity. The authors also investigated a potential interaction of the treatment with standard immune suppressive medication but could not detect any effects on the blood levels. [95].

Contrary to the limited number of clinical studies, a variety of preclinical studies observing promising results exists. In a meta-analysis by Wang et al. [96] 18 studies investigating the effects of curcumin in animal IRI models involving 396 animals were evaluated. The authors observed anti-inflammatory, antiapoptotic as well as antifibrotic effects. Similar results were obtained in the case of quercetin. In animal models, a decrease of oxidative stress after IRI was observed [91,97,98,99], reflected by lower levels of thiobarbituric acid reactive substances (TBARS) [100] and MDA [101] as well as an increased activity of antioxidant enzymes [101]. Additionally, the inhibition of IRI-induced inflammation [91,99] and apoptosis/ferroptosis [99,102], as well as the induction of autophagy [103], finally resulting in decreased tissue damage, and improved organ function has been observed [97,98,102].

### 5.2. Liver

Analogical to its use in heart transplantation, quercetin has not been investigated in the clinical setting of liver transplantation. However, a variety of preclinical studies have shown its putative benefits in hepatic IRI. Thereby, improved plasma levels of liver enzymes AST, ALT [98,104] and the amelioration of oxidative stress [104,105], along with the inhibition of apoptosis, autophagy [106] and attenuation of liver injury [98,105], have been reported. Moreover, quercetin synergistically increased the protective effects of remote ischemic reperfusion preconditioning [98], as well as of the Fe^2+^ chelator deferoxamine [107]. 

The situation for curcumin is similar. So far, no clinical studies on its protective effects against IRI during liver transplantation have been conducted. Contrary, a plethora of preclinical studies showing very promising results exists. In animal models, curcumin could prevent liver injury during IRI [108,109,110,111]. This was reflected by the attenuation of liver enzymes AST, ALT [112,113] and restoration of the ATP content [110]. The tissue protective properties of curcumin were accompanied by anti-inflammatory [108,113,114,115] and protective effects against oxidative stress [111]. Interestingly, the anti-inflammatory effects have also been observed for remote lung injury [112]. Moreover, the addition of curcumin into a preservation solution has been shown to protect against oxidative stress and histological injury after cold [116] as well as warm [116] storage. Finally, the protective effects of curcumin culminated into the increased post-transplantation survival of treated animals. 

### 5.3. Heart and Vessels

Although no clinical studies on quercetin concerning IRI in the heart SOT setting appear to be available in online databases, a variety of preclinical studies has been conducted. The utilization of quercetin in the course of IRI maintained cardiac health, as implicated by the improved cardiac flow [117], alleviated cardiac dysfunction [118,119,120] and injury [119,121], as well as decreased infarct sizes [119,122]. On the molecular level, the inhibition of apoptosis [118,119,120,122] and inflammation [119,120,123], as well as the amelioration of oxidative stress [120] and improved mitochondrial function [124], have been observed. Interestingly, several studies found differences in the effectiveness in terms of recipient age and metabolic conditions, as well as the duration of application. Accordingly, application during reperfusion yielded improved protection compared to the application before reperfusion [117]. Moreover, advanced protection has been reported in case of juvenile animals [125]. Thus, the benefits were lower in the case of diabetic and old animals. The authors speculated about an impaired activation of the PI3K/Akt axis as the underlying cause [126].

Contrary to quercetin, one clinical study addressing the protective effects of curcumin on myocardial IRI has been conducted. The authors observed significantly less postoperative myocardial infarctions in patients undergoing CABG. Additionally, curcumin could decrease the inflammatory state, as reflected by reduced levels of CRP. Moreover, reduced levels of MDA pointed towards ameliorated oxidative stress [127]. 

The outcome of this clinical study went along with various preclinical studies showing the restoration of cardiac function [128,129,130], as well as the attenuation of cardiac lesions [131,132,133]. Moreover, the amelioration of cold storage-induced myocardial damage by the use of curcumin has been reported [134]. These results are not surprising, as curcumin has been shown to inhibit apoptosis [128,132,135] and inflammation [132,136] and to ameliorate levels of oxidative [132,137,138] and endoplasmic reticulum (ER) stress [132,137] in various preclinical models of myocardial IRI. 

### 5.4. Concluding Remarks

Curcumin and quercetin share a low incidence of clinical studies conducted. Only one clinical study combined both flavonoids for their use in kidney transplantation. For liver and heart transplantation, no clinical studies utilizing curcumin and/or quercetin have been conducted so far. However, one study investigated the effects of curcumin on IRI during CABG. Both studies could observe beneficial effects on the affected organ. These positive results were underpinned by a literal plethora of preclinical studies (18 animal studies on curcumin in kidney IRI) showing promising results. Additionally, preclinical studies investigating quercetin observed beneficial effects. Interestingly, a better protection by quercetin has been observed in young animals compared to older and diabetic ones. As the PI3K/Akt pathway is a key target of quercetin, the missing effect was explained by an impaired PI3K/Akt in the latter conditions. This should be kept in mind while planning future clinical studies. 

To sum up, both polyphenols share a long tradition of usage, low costs and toxicity. Together with the promising antioxidative, antiapoptotic and anti-inflammatory properties, among the others observed in clinical, as well as preclinical, studies, this warrants further investigations in the clinical setting.

## 6. Glycine

Glycine is a nonessential proteinogenic amino acid. With regards to IRI, glycine has a special role, since the mechanisms by which the amino acid seems to be involved in IRI prevention are not fully understood. In 2011 a German working group wrote a review paper with the appropriate title "*Glycine, a simple physiological compound protecting by yet puzzling mechanism(s) against ischaemia-reperfusion injury: Current knowledge*". The means discussed therein included aspects of the direct cytoprotection and inhibition of the inflammatory response. In both overarching categories, there are now numerous elucidated molecular biological mechanisms of glycine that (could) contribute to IRI. For a deeper understanding of these mechanisms, we recommend the reader to take a look at the mentioned publication [139]. It should be stated, though, that the protective mechanisms of glycine do not seem to depend on its metabolism [140].

### 6.1. Kidney

The scientific evidence for glycine in renal IRI prevention is modest. We found no clinical study directly dealing with this topic, although there were a few experimental studies in the animal model: Glycine as part of preservation medium was shown to be protective in the hypoxic injury of rabbit renal tubules [141]. It also appeared to stabilize overall membrane integrity (and presumably membrane tertiary structure) as part of a perfusion medium of isolated rat kidneys. Importantly, the effect was not blunted by blocking glycine metabolism This suggests that the protective effect of glycine does not depend on its metabolism, which is in line with more recent studies (review papers mentioned in the beginning of the section) [140]. Glycine, as part of reperfusion medium, has also been shown to be membrane stabilizing and cytoprotective in canine derived cold-stored renal tubules. Yet, the addition of glycine had no effect on ATP regeneration of mitochondrial functions [142]. The cytoprotective and membrane stabilizing potential of glycine investigated in a cell culture model of renal anoxia/reoxygenation injury showed some protection albeit lower than authors had expected [143]. The latest two studies show conflicting results. Yin et al, found that glycine as a bolus injection and subsequent dietary supplementation significantly reduced cellular damage, which culminated in improved glomerular filtration. [144] However, Arora et al. observed a deterioration in all investigated parameters after intraperitoneal administration of glycine to rats [145]. A possible explanation for the discrepancy might lie in the different ischemia times being 40 minutes in the study by Arora et al, compared to only 15 minutes in the study by Yin et al. In the study by Yin et al, no improvement was seen with prolonged ischemic episodes. 

### 6.2. Liver

As mentioned, the molecular biological mode of action of glycine in the context of IRI is complex. Apart from the publication by Petrat et al. [139], there are two more review papers that solely focus on the key mechanisms of glycine in hepatic IRI prevention [146], [147]. An important hallmark of glycine’s hepatic IRI prevention is its inhibition of Kupffer cell activation [148]. Additional details are published in the work by Liu et al. [149]. Despite the sound available literature on the mechanism of action of glycine in preventing IRI and the relatively high number of conducted animal studies with quite positive outcome, the number of performed clinical studies remains low. We only found four clinical studies dealing with glycine in the prevention of hepatic IRI, three of them are in line with the research by Habib et al. [147] published 15 years ago. Two of these noteworthy small studies (*N* = 4, and *N* = 7, respectively), both conducted by the same person, used a 300 mM glycine infusion 1 hour before reperfusion. In both trials, serum ALT and AST levels were markedly lower during the first week after transplantation compared to matched controls [150,151]. One of the studies also reported a higher incidence of primary graft dysfunction in the control group not receiving glycine infusion [150].

The third trial conducted by Arora et al., (RCT, *N* = 50) assessed the effect of hepatic arterial and portal veins flush with a 2 mM glycine solution before reperfusion. The authors noted a significant reduction of serum ALT for 3 days post orthotopic liver transplantation. Moreover, the occurrence of ischemic type bile duct strictures decreased compared to the control group [152]. The most recent and fourth study we found dates back to 2005. Unfortunately, we only found its published study protocol [153]. This is a pity, as this study seems to be a RCT in terms of its setting and appears to be the largest study on the subject to date. The assessed endpoints included levels of AST and ALT, as well as graft and patient survival up to 2 years after transplantation. Glycine has been administered during the anhepatic phase, as well as after liver transplantation. The only published results we were able to find was an online version of a meeting abstract of the 2016 American transplant congress [154]. Interestingly, the abstract stated that glycine treatment has not induced significant alterations neither in the primary nor the secondary endpoint. However, there appeared to be a non-significant reduction of the ALT levels, as well as an improvement of patients’ overall survival in a sub-group analysis of patients with higher plasma levels of glycine. Moreover, significant reductions in both mild and moderate IRI have been observed in this subgroup. The abstract additionally suggested that glycine also positively influenced kidney function reflected by estimated glomerular filtration rate (eGFR) during cyclosporine treatment.

### 6.3. Heart and Vessels

For the application of glycine in terms of cardiac IRI prevention, no clinical studies addressing this topic could be found. However, a few studies in animal models have been conducted. A study in rats investigated the effect of dietary glycine on chronic rejection of aortic allografts. Glycine prevented dramatic intimal thickening and medial thinning as observed in allografts from rats fed control diet. In addition, infiltrating macrophages were significantly reduced in the adventitia in animals fed glycine. [155].

Warnecke et al., used a porcine heart transplantation model to investigate glycine on cardiac IRI prevention. Hearts of glycine-treated donors could be loaded with a significantly increased volume after 1 hour and after 2 hours of reperfusion but maximal right ventricular developed pressures remained unaffected. Postischemic right ventricular end-diastolic compliance was significantly higher in glycine-treated animals, concluding that glycine exerts useful, albeit modest effects in heart transplantation [156]. 

### 6.4. Concluding Remarks

Although Glycine is well-acknowledged for its IRI-protective effects, only a limited number of studies investigated this compound in the context of IRI during SOT. Especially clinical studies are very scarce and can only be found in the context of liver SOT. Although these clinical studies were performed with a very limited number of participants, the majority reported about beneficial effects on graft health. The positive results observed in clinical studies are backed up by a variety of preclinical studies especially addressing hepatic IRI. Glycine seems to be especially beneficial for this setting as pleiotropic effects protecting liver tissue, e.g., by inhibiting Kupffer Stern cells have been observed. Besides that, several preclinical studies can be found in the context of IRI in kidneys. Interestingly, one preclinical study reported about negative results. In this study, longer periods of ischemia have been introduced. If this is the reason for the negative consequences and may even apply for other organs as well remains to be investigated. However, the vast majority of studies observed positive results. One study even reported about beneficial effects of dietary Glycine. If this will be verified, the administration will be very easy and can also be dedicated for extended outpatient use. Along with its easy availability and low-cost, Glycine is considered an important candidate for future clinical trials.

## 7. Omega-3 Polyunsaturated Fatty Acids

Marine omega-3 polyunsaturated fatty acids (n-3 PUFA) have been appreciated for their anti-inflammatory effects for a long time. The anti-inflammatory effects are mediated by replacement of arachidonic acid in cellular membranes. This process is crucial for inflammatory balance as the n-3/n-6 ratio released from phospholipid membranes determines the fate of subsequently formed eicosanoid mediators. Whereas release of mostly arachidonic acids mainly leads to formation of proinflammatory mediators like prostaglandins or thromboxanes, the release of eicosapentaenoic acid (EPA)/docosahexaenoic acid (DHA) results in formation of anti-inflammatory mediators like resolvins or protectins. The latter are known to support the resolution of an inflammatory process by inhibiting proinflammatory cytokines, limiting neutrophilic infiltration and enhancing macrophagic resolution [157]. Beside their well-known anti-inflammatory activity, n-3 PUFA possess antioxidative activities as well. Interestingly, the anti-oxidative effects are induced via formation of free-radical-generated oxidation products rather than by direct antioxidative effects. n-3 PUFA are even more prone to oxidation compared to arachidonic acid as a consequence of their higher number of double bonds. The formed oxidation products subsequently bind the inhibitor of Nrf2, thereby activating the well-known Nrf2-pathway to activate the endogenous antioxidative protection mechanisms [158]. Accordingly, the induction of heat-shock proteins has been observed upon n-3 PUFA treatment [159].

### 7.1. Kidney

Especially in the field of kidney transplantation plenty of clinical studies have been conducted showing contradicting results. Tatsioni et al. [160] performed a meta-analysis of 16 RCTs comprising 812 patients. Some studies observed beneficial effects on renal function and on recovery after rejection periods. Additionally, improvement of filtration fraction and glomerular filtration rate (GFR) response after amino acid load have been observed in a subset of patients. However, studies were characterized by high heterogeneity based on different inclusion criteria, outcome measures and amount of utilized treatment medication and did not reveal significant effects on recovery of renal function or prevention of acute rejection. Interestingly, the same set of studies apart from minor changes has been investigated in the course of a Cochrane review by Lim et al. [161]. Similar conclusions have been drawn, stating that available data do not unequivocally demonstrate renal protection in the course of organ transplantation. In contrast to clinical studies, beneficial effects of marine n-3 PUFA treatment on renal IRI has been observed in animal studies. This was based on preserved tubular epithelial cell integrity [162], decreased levels of oxidative stress [163,164] and inhibition of apoptotic pathways [164] finally leading to less histologic damage [163] and improved renal function [165]. 

A fundamental difference between these clinical and animal studies is the timepoint of application. In animal studies, n-3 PUFA were administered for 2 weeks [162,163,164] up to 12 weeks [165] prior to IRI. The majority of clinical studies started the treatment only after completed transplantation (as reviewed in [160]). It has been criticized that this might be too late as supplementation with n-3 PUFA takes at least three weeks for sustained effects on cytokine levels [166]. Alexander et al. [167] described that serum levels of EPA/DHA after n-3 PUFA supplementation did not reach its peak before 30 days of treatment. This might also explain the outcome of three more recent studies. Patients were treated with a delay of 6 to 8 weeks [168,169] post transplantation or immediately after transplantation but only for a few days [20]. These studies could neither observe protective effects on renal function [20,168] nor on quality of life [169]. Indeed, a study administering n-3 PUFA immediately after transplantation for a longer period found raised blood levels of EPA/DHA which were correlated with reduced events of rejection. However, in this study L-arginine was also used and synergistic effects cannot be excluded [167].

### 7.2. Liver

The administration of n-3 PUFA in liver transplantation was firstly described in 1995 in the context of immunonutrition [170]. Since then, a large number of clinical studies had been conducted. Lei et al. performed a meta-analysis of 4 RCTs involving 188 patients and found improved liver function reflected by decreased AST but not ALT or total bilirubin. Furthermore, no significant effects on mortality or rejection episodes upon treatment with n-3 PUFA have been observed [170]. Two Chinese studies by Zhu et al. utilizing n-3 PUFA also observed improved liver function reflected by decreased ALT [171,172]. Similar to the meta-analysis of Lei et al., other parameters of liver function like AST, total bilirubin or LDH had not been altered in the course of n-3 PUFA treatment [171]. However, contrary to Lei at al., dampened hepatic cell injury, as well as reduced portal inflammation, have been observed [171,172]. Furthermore, the duration of hospital stays was significantly shorter in the treatment group [171]. Another Chinese study investigated the impact of n-3 PUFA on IRI during liver resection. Similar to the studies of Zhu et al., patients were treated intravenously several days post transplantation. The authors observed a lower degree of inflammation and protection of liver function as seen by decreased levels of white blood cells (WBC), as well as AST, ALT and total bilirubin, respectively. Furthermore, lower frequencies of complications, as well as decreased hospital stay duration, could be observed [157]. Ibrahim et al. [173] looked at the other side of the coin investigating the beneficial effects of n-3 PUFA supplementation on donor liver recovery after resection for a scheduled living donor transplantation. Positive effects on inflammation and liver function were observed as seen at reduced levels of WBC and CRP, as well as AST and ALT, respectively. Moreover, an increased regeneration index, in addition to increased levels of the hepatic growth factor, have been observed. Along with the latter, increased donor liver volumes have been observed. Three studies addressing the effects of n-3 PUFA in the context of hepatic ischemia-reperfusion injury utilizing n-3 PUFA in combination with L-arginine and ribonucleic acid observed contradicting results. Plank et al. conducted a pilot open-label study as the control group was of historic nature. Oral application for a median of 54 days did neither impact ICU stay duration nor rejection event frequencies [174]. A larger-scale follow-up study by the same authors and the same setting could not find beneficial effects on duration of hospital stays, complications or rejection episodes either [175]. The third study from Switzerland investigated the effect of this combination on patients undergoing open liver surgery or liver resection. Compared to the previously mentioned studies, patients were treated intravenously with n-3 PUFA, L-arginine and ribonucleic acid only twice. The authors did not observe beneficial effects on complications or mortality rates, respectively. Additionally, the subgroup analysis for patients experiencing inflow occlusion did not reveal beneficial effects [176].

Why the combination studies could not observe protective effects during hepatic IRI remains to be elucidated. Linecker et al. [176] argued, that the previously mentioned studies administered n-3 PUFA parenterally for several days and that this procedure would be harmful to liver function. The administered n-3 PUFA would attenuate these detrimental effects. This phenomenon would be circumvented by oral administration as performed by the majority of n-3 PUFA combination studies. In order to prove this claim, further studies comparing oral or parenteral administration of n-3 PUFA will be necessary. However, results of animal studies give rise to doubt that the route of administration is the reason for a lack of positive results. In animal studies, oral [177] and parenteral [178] administration of n-3 PUFA lead to hepatic tissue protection [177,179,180] and anti-inflammatory effects [180,181] upon IRI. 

### 7.3. Heart and Vessels

To the best of our knowledge, no studies on the protective effects of n-3 PUFA on ischemia-reperfusion injury during heart transplantation have been conducted. However, beneficial effects in the setting of coronary artery bypass grafting have been observed in two studies. A study by Veljovic et al. observed accelerated return to aerobic metabolism after ischemia-reperfusion injury. This was reflected by faster clearance of lactate and fast onset of O_2_ consumption. Moreover, lower levels of myocardial tissue damage have been observed. [159] The second study investigated n-3 PUFA in combination with vitamin C and vitamin E in patients undergoing coronary artery bypass or valve surgery. Oral treatment for 7 days lead to significantly reduced levels of biomarkers indicative of oxidative stress and inflammation. This was accompanied by increased activities of endogenous antioxidant enzymes and inhibition of prooxidant enzymes. Finally, the authors observed a significantly lower occurrence of atrial fibrillation. [182] The latter condition is strongly associated with ROS generated during IRI. The modulations induced by ROS ultimately culminate into increased intracellular levels of calcium. This is especially dangerous for the cardiac muscle, as it regulates its contractile activity and may cause atrial fibrillation. The specific beneficial effects against atrial fibrillation observed by n-3 PUFA may arise from their ability to incorporate into cellular membranes and thus regulate cellular calcium influx and consequently cardiac contractile activity [158].

Contrary to a lack of clinical studies, several animal models utilizing n-3 PUFA against IRI in the course of heart transplantation have been investigated. Thereby, positive effects on cardiac graft survival [183,184] have been reported. As underlying mechanisms, anti-inflammatory effects [183,184,185] have been discussed. 

### 7.4. Concluding Remarks

n-3 PUFA have been appreciated for their anti-inflammatory effects for a very long time. Thus, it is not surprising that a plethora of clinical studies has been conducted. This is especially true for kidney SOTs. Unexpectedly, ambiguous results have been observed. These results are contradicted by a variety of preclinical studies observing beneficial effects. It has been claimed that in clinical studies, n-3 PUFA have been administrated too late and for too short period of time. Interestingly, the majority of studies addressing liver SOT reported about positive results although n-3 PUFAs had been administered only after transplantation and only for short periods of time. No clinical studies have been conducted in the context of heart transplantation, why they were substituted by CABG or valve surgery studies. These studies could observe beneficial effects as well and especially emphasized on the antioxidative potential of PUFAs. If the lack of positive results in kidney studies is associated with administration period or timing or is especially linked to the organ itself, remains to be elucidated. However, the easy availability and the vast majority of promising data encourage the conductance of future clinical trials elucidating the full potential of this compound. 

## 8. N-Acetyl Cysteine

Strictly speaking, N-Acetyl Cysteine (NAC) is not a micronutrient in the classical sense, as the substance does not occur in nature and thus has to be produced synthetically. However, since it is the derivative of the naturally occurring amino acid L-cysteine, a crucial compound in the food supplement industry due to its attractive properties and a very well investigated compound in the context of IRI during SOT and other conditions we have decided to include NAC in this compilation. NAC is an N-acetyl derivative of the amino acid L-cysteine, which, by increasing glutathione, exerts an indirect but strong antioxidant effect. NAC is characterized through its high solubility in water, good tolerability, and broad availability through its inexpensiveness.

### 8.1. Kidney

The clinical study situation regarding IRI prevention by NAC in the kidney is modest and controversial. Our literature search has revealed a hand full of clinical studies directly dealing with the influence of NAC on the success of kidney transplants and/or IRI. Interestingly, almost all of them share a similar dose and route of administration, being 600 mg NAC twice a day orally for various time periods. 

In 2008, Fuentes et al. tested whether 600 mg NAC (oral, twice a day) had an effect on oxidative stress markers, serum lipids, and renal function in 25 patients with stable renal function after transplantation. While there were no significant differences in oxidative stress markers before and after treatment with NAC, the mean serum high density lipoprotein (HDL) levels increased while showing a significant positive correlation with GPx. Serum creatinine and related Cockcroft-Gault formula improved significantly. The authors concluded that NAC treatment in patients with stable renal function after transplantation had a positive influence on renal function [186]. 

Danilovic et al., investigated the effect of oral NAC (600 mg twice daily) on early outcomes of deceased donor renal transplantation. The treatment group receiving NAC, showed a lower mean serum creatinine during the first 90 days and 1 year after transplantation, as compared to the control group not receiving NAC. Oxidative stress as measured as serum levels of TBARS was significantly attenuated within the NAC group. Furthermore, the NAC group showed a higher Cockroft-Gault eGFR throughout the first 90 days and 1 year after transplantation. Accordingly, delayed graft function was rarer among the NAC group allowing the NAC recipients to undergo fewer dialysis sessions [187].

An Iranian RCT conducted on 50 deceased-donor renal transplant recipients investigated the effect of 600 mg (oral) NAC twice daily on graft function and renal tubular injury. The group receiving NAC showed significantly lower mean serum creatinine levels during the first 90 days as well as 1 year after the transplantation, highlighting the potentially far-reaching benefits of perioperative supplementations. The authors concluded that NAC has a promising potential in reducing tubular injury and improving graft function, evidenced by significant reduction in the rate of reduced graft function (RGF) and levels of plasma neutrophil gelatinase associated lipocalin (p-NGAL) [188].

Two studies from 2015 failed to confirm previous successes from clinical and/or animal studies: The first study, carried out by Orban et al., examined the effect of NAC pretreatment on deceased organ donors on renal allograft function. Here, the donors were administered 600 mg NAC 1 hour before and 2 hours after cerebral angiography (to confirm the brain death of the donors) intravenously in a single-blinded controlled fashion. Delayed graft function was used as the primary endpoint, measured by the need for at least one dialysis session within the first week after the transplantation or a serum creatinine level higher than 200 µmol 7 days after the transplantation. There were no differences in either of the two parameters recorded [189]. 

The second study shared the same primary endpoint (delayed graft function) but assessed it through the urinal biomarkers neutrophil-associated lipocalin (NGAL) and interleukin-18 (IL-18). The graft recipients allocated to the control group received routine antirejection medication, while the NAC group got three doses of NAC 600 mg (6 hours before grafting and 12 and 18 hours after transplantation) added to the immunosuppressive regimen. This study found reductions in NGAL and IL-18 compared to the control group, but these lacked statistical significance [190]. 

There are other clinical studies examining IRI in the context of NAC and the kidney, but not in the context of a kidney transplant. The work of Burns et al. for example has attempted to combat renal dysfunction as a consequence of IRI triggered by coronary artery bypass graft surgery. In this study, trends and slight differences in terms of need of replacement therapy, serious adverse events, hospital mortality, or length of hospital stays, could be measured, yet lacking statistical significance [191]. A Korean study investigating the protective effects of NAC on acute kidney injury in high-risk patients undergoing off-pump artery bypass also failed to demonstrate any statistically significant effects: Perioperative administration of NAC did not prevent the development of postoperative acute kidney injury after coronary artery bypass graft surgery [192]. A Brazilian trial investigated the very same endpoint, randomizing patients undergoing off-pump coronary bypass graft surgery into groups receiving nothing or NAC 600 mg twice a day. Their perioperative intervention had no effect on acute kidney injury and associated morbidity and mortality [193]. 

One small cross-over study (n = 11) from 2009 that did not investigate effects of oral NAC, but intravenous NAC found it to be highly effective in reducing plasma total homocysteine levels in renal transplant recipients. Even though the study did not claim to directly measure consequences of transplantation-associated IRI, hyperhomocysteinemia can be considered as a marker of renal dysfunction and as such reflect protective effects on transplantation-associated IRI [194].

### 8.2. Liver

Jegatheeswaran et al., reviewed the experimental and clinical evidence for modification of hepatic IRI by NAC in 2011 [195]. Upon analyzing 40 studies, the authors concluded that (i) clinical studies using NAC were almost uniformly of poor quality and that (ii) NAC, administered before induction of liver IRI in experimental (animal) models, may have a positive effect on markers of liver injury, but that there was little evidence that this effect translated in a positive fashion to any clinically relevant endpoint, neither in liver transplantation nor in liver resection surgery.

Another albeit smaller review from 2008 drew similar conclusions [196]. Since then, only a hand full of additional clinical trials investigating NAC on liver IRI had been conducted. In 2013, one study assessed the effect of NAC administered through the portal vein (300mg) 1 hour before liver procurement just before cross clamping. In this setting, NAC was able to significantly improve graft survival after liver transplantation with respect to the group not receiving NAC. The study however did not find any significant changes in hepatic biomarkers such as ALT, AST or bilirubin on any postoperative timepoint except for AST on postoperative day 15, favouring the NAC group [197]. 

Within the same year, a study investigating patients undergoing elective hepatic resection failed to demonstrate noteworthy effects through perioperative administration (infusion, 10 g/24h) of NAC. NAC had no influence on reduction of hepatocellular injury as measured by serum ALT levels. Overall, NAC-treated patients experienced a higher frequency of post-hepatectomy liver failure (predominantly of grade A). There was a trend towards a lower incidence of clinical post-hepatectomy liver failure (grades B&C) and overall complications in NAC-treated patients. Yet, no clinical advantage of significance could be observed in the study [198]. 

Aliakbarian, et al. tested whether NAC would affect the severity of IRI in orthotopic liver transplant in adults when added to the University of Wisconsin (UW) preservation solution. Within the scope of the study, 2 g of NAC were added to 1 litre of UW solution within the experimental group while the standard UW solution was used in the control group. A donor liver was then perfused with the respective solution via the superior mesenteric vein. The double-blind, RCT showed no significant differences between the groups in terms of time to hepatic artery reperfusion, hospital stays, vascular complications, inotrope requirements before and after the portal vein was de-clamped, and blood gas analysis [199]. 

Apart from the small number of clinical studies since 2011, further investigations were carried out in animal models. Like those pre-2011 animal studies, these showed consistently impressive successes too. 

### 8.3. Heart and Vessels

Most of the studies looking at NAC and IRI in the heart focus on bypass surgery instead of transplants. In this regard, however, the quantitative study situation is exceptional: In 2019, a systematic review was published which evaluated the effects of NAC on IRIs in cardiac surgeries in general. The study, included 29 RCTs comprising 2486 participants, examined, among others, mortality, acute cardiac insufficiency, and length of hospitalization. The meta-analysis finally showed favorable effects for NAC for the majority of the parameters, even if not entirely significant in total. Statistical significance as depicted in a forest plot was achieved for length of hospitalization, but only when NAC was added to the cardioplegia solution, not when administered orally [200]. 

Apart from the question of general cardiological interventions, we were only able to find one clinical study dealing with cardiac transplant surgery albeit there was no mention of IRI, and we were unable to access the full text study. According to the title of the respective study, NAC was not able to lower plasma homocysteine concentrations and not able to improve brachial artery endothelial function in cardiac transplant recipients [201].

In terms of heart transplants, there is some literature available in animal models. A study performed in rats demonstrated impressive effects of NAC in terms of IRI prevention in cardiac transplantation. The group of animals that received NAC showed improved organ function reflected by significantly improved shortening of isografts and decreased serum LDH in addition to improved markers of inflammation [202]. In another rat study, NAC was able to improve serum levels of LDH in addition to liver markers AST and ALT. Furthermore, amelioration of oxidative stress in addition to attenuated apoptosis has been observed [203].

### 8.4. Concluding Remarks

There has been a broad research interest in investigating the beneficial effects of NAC on IRI during SOT. Hence, an enormous number of studies has been conducted, albeit showing contradicting results. A possible explanation for this ambiguity could be the high heterogeneity in terms of route of administration and clinical endpoints. Interestingly, there are also large differences in the outcome between the different organs investigated. Whereas the study situation in the kidney is controversial, some studies in the liver found even negative effects. Compared to that, NAC administration during cardiac IRI showed consistent positive results that are backed up by preclinical transplantation models. However, it should be mentioned that so far clinical studies only addressed IRI during bypass surgery rather than cardiac transplantation. These results again point out the importance of standardized trial designs in order to be able to better compare the single studies. Despite the partly controverse findings, NAC appears to have potential for a good candidate combating IRI. However, future studies should elucidate if beneficial effects of NAC depend on the route of administration and are restricted to certain organs. Additionally, the clinical endpoint should be selected carefully in order to receive a representative picture.

## 9. Resveratrol

Resveratrol was first isolated from roots of white hellebore (*Veratrum grandiflorum*) in 1940. Since 1963 it was isolated from the roots of *Polygonum cuspidatum*, which is also used in Chinese/Japanese medicine. [204] Resveratrol belongs to the class of stilbene [205] and can be found in certain foods like peanuts [205,206], grapes [205,206], some roots [206] and berries [205,206]. In plants, it is synthesized as response to environmental stressors [205] like fungal infections, UV-radiations and cold [207]. Resveratrol has been associated with the “French paradox”. This phenomenon was termed after the French population who has been known for their red wine consumption. Interestingly, the red wine consumption has been linked to a lower incidence of cardiovascular diseases in the French population [208].

Resveratrol targets very crucial master regulators like SIRT-1 or AMP-activated protein kinase (AMPK) [207]. The former is a known regulator of transcription, apoptosis and metabolism [206] and the latter is a regulator of metabolism as well. By targeting these master regulators amongst others, resveratrol has been recognized for its anti-inflammatory [205], anti-oxidative [209], anticarcinogenic [206,209], endothelium-protective [209] and antiapoptotic properties, in addition to its ability to induce autophagy. These effects finally translated into reduction of hypertension [205], and increased insulin sensitivity [205] as well as improved lipid levels [209]. Interestingly, resveratrol has been shown to mimic the effects caloric restriction [206]. For this reason, it is acknowledged for its antiaging effects and its ability to increase lifespan has been reported in in vitro and animal studies with vertebrates [206]. 

### 9.1. Kidney

To the best of our knowledge, no clinical study investigating the beneficial effects of resveratrol in the context of IRI in kidney transplantation has been conducted so far. Contrary to the lack of clinical trials, a large number of animal models investigating this compound exists. One animal model investigated the effect of resveratrol on kidney autotransplantation in pigs. Thereby, resveratrol has been added to the preservation solution in a static, as well as machine perfusion approach. Improved glomerular filtration and proximal tubular function, as well as the slowing of chronic loss of function and the onset of histological lesions, has been observed for the latter. The use of resveratrol in the preservation solution for machine perfusion led to a decrease of oxidative stress and decreased levels of apoptosis, as well as slowed chronic loss of function, in addition to interstitial fibrosis and tubular atrophy [210]. Besides this animal model specifically dealing with transplantation, several other animal models addressed the protective effects of resveratrol in different settings of IRI. The administration of resveratrol reduced mortality [211] and improved renal function [212,213] reflected by the attenuation of increased serum urea [213], in addition to reduced creatinine levels [211]. Additionally, protective effects of the level of morphology have been observed as well [211,214]. These included the preservation of glomerular number reduction [213] and collapse [215]. The observed beneficial effects on kidney tissue were associated with inhibited inflammation [212], and decreased oxidative stress [212,213,214,216,217], reduced apoptosis and necrosis [212] as well as induction of autophagy [216] in the course of IRI. In contrast to the previously mentioned studies observing protective effects, two animal studies are contradicting showing no effect [217]. These differences could be explained by the way of application, as resveratrol was administered immediately before induction of ischemia in contrast to the other studies administering it for days or at least 40 minutes before inducing ischemia. However, if this is really the case has to be investigated in further studies.

### 9.2. Liver

To the best of our knowledge, no clinical study investigating the beneficial effects of resveratrol in the context of liver transplantation has been conducted so far. Nevertheless, the influence of SIRT1 expression, a well-known target of resveratrol, has been investigated in human liver grafts. Thereby, the observed correlation between SIRT1 levels and improved hepatocellular function gave first hints about the beneficial effects of resveratrol in the clinical setting [218]. Additionally, there is sound evidence of resveratrol showing beneficial effects in various animal models. 

The utilization of resveratrol in animal models had a positive impact on liver preservation. At the functional level this has been reflected by decreased levels of plasma aminotransferase activities [219], improved bile production [220] as well as increased portal vein flow volume [220]. Additionally, attenuation of IRI-induced thioredoxin-interacting protein (Txnip) indicative of liver stress has been reported [221] along with increased ATP-content of liver grafts [220]. Moreover, resveratrol treatment induced positive effects on a morphological level including preservation of sinusoidal endothelial cells [220] along with generally reduced histological liver injury [219]. The beneficial effects finally prolonged the survival of treated animals [222]. Several publications traced these beneficial effects back on decreased oxidative stress [219,222,223,224] anti-inflammatory effects [218,220] as well as ameliorated cell death [223]. Decreased markers of oxidative stress [222,223] were observed to be a consequence of attenuated HIF-1α upregulation [224] in addition to upregulated endogenous antioxidant systems comprising GPx, CAT and SOD [219]. Interestingly, the inhibition of apoptosis and necrosis was found to be mediated via stabilization of mitochondrial membranes [223]. Finally, it should be mentioned that the study conducted by Hassan Khabbar et al. reported about concentration-dependent effects of resveratrol on liver health: while resveratrol at low concentration has been found to promote liver protection as a consequence of antioxidative effects, it became prooxidant and even aggravated liver injury at high concentrations. [225] The investigation of proper dose range should therefore be kept in mind while planning clinical trials. At this point, we would like to stress that it pays to look at resveratrol in its entire role in liver diseases and hereby recommend the excellent and very recent review by Izzo et al. [226].

### 9.3. Heart and Vessels

Although, the beneficial effects of resveratrol on cardiac health are well known, no clinical studies addressing the protective effect of resveratrol on IRI in the course of heart transplantation have been published yet. However, a decent number of preclinical studies investigated its effect on heart health during IRI. Thereby, multiple beneficial effects have been observed. The administration of resveratrol led to attenuated cell death as reflected by reduced infarct size [227,228,229,230,231], reduced apoptosis [227,229] and reduced levels of tissue integrity markers LDH [230,231,232] and CK [231,232]. Moreover, in an ex vivo model, alleviated myocardial injury has been observed as well [233]. Along with antiapoptotic effects, beneficial effects on the level of oxidative stress [232,233] as well as anti-inflammatory effects [229,230,231] have been demonstrated. Besides these systemic protective effects, resveratrol specifically provided beneficial effects for the cardiovascular system, as it improved the endothelial in an animal model of myocardial infarction [227,228,231]. The sum of all protective effects observed culminated in improved cardiac function [228,232] in addition to reduced rhythm disturbances [231].

### 9.4. Concluding Remarks

Although resveratrol is a well-established antioxidant, it has not been used in clinical studies on IRI during SOT so far. Yet, as known activator of SIRT1 its potential beneficial contribution in SOT-induced IRI has been pointed out by a clinical study reporting about a correlation between upregulation of SIRT1 and improved liver function. A large number of animal models observing protecting effects supports this hypothesis. Some of the protective effects specifically targeted heart health like increase of VEGF or p-eNOS. This was less surprising, as resveratrol is a known compound supporting heart health associated with the French paradox. However, in kidney and liver models some limitations like a lack of effect of immediate application or negative effects upon use of too high concentrations were observed as well. These important lessons from animal models should be kept in mind for future clinical trials.

## 10. Sulforaphane

Sulforaphane is an isothiocyanate phytochemical found in cruciferous vegetables such as broccoli or brussels sprout. Through binding the Nrf2 repressor protein kelch-like ECH-associated protein 1 (Keap1), sulforaphane exerts a strong indirect antioxidative effect. As activated Nrf2 translocates into the nucleus and binds to the antioxidant response element (ARE) promotor regions, transcription of important cytoprotective and antioxidative genes (transcription products of these genes are often referred to as phase 2 enzymes) including NAD(P)H:quinone oxidoreductase 1, carbonyl reductase 1 (CBR1), HO-1, or GPx is initiated. In addition, some in vitro, in vivo, and preliminary clinical studies have demonstrated chemopreventive and tumor-suppressive properties of sulforaphane through multifaceted mechanisms.

### 10.1. Kidney

Our literature research did not reveal any clinical studies investigating the beneficial effects of sulforaphane on kidney function in the context of IRI in SOT.

Contrary to clinical studies, various in vitro and animal studies reported about the beneficial effects of sulforaphane on renal IRI. First hints came from an in vitro study that reported about reduced cytotoxicity and induction of phase 2 enzymes after the application of sulforaphane in the course of hypoxia-reoxygenation [234]. These results could be confirmed in vivo showing reduced renal injury reflected by improved histopathologic scoring [234] and improved kidney function [235]. Not surprisingly, the beneficial effects on organ preservation were traced back to anti-inflammatory as well as anti-apoptotic effects and were even more effective than the well-known ischemic pre-conditioning [235].

A group led by P. Schemmer in 2013 confirmed beneficial effects of sulforaphane on renal IRI prevention. In their trial, rats that received sulforaphane 24 and 1 hour before and 6 hours after transplantation showed significantly lower blood urea nitrogen levels after kidney transplantation as compared to the control animals receiving saline. Serum creatinine levels were significantly decreased in the sulforaphane group 24 hours after transplantation. Importantly, the researchers found a significantly better preservation of mitochondrial microstructure in sulforaphane-treated animals, which was in line with significantly increased SOD2 gene expression. The findings could be confirmed on histopathological scale, as sulforaphane treatment led to a decrease of severely injured tubules [236].

So far, modulation of the Nrf2 pathway via sulforaphane appears a promising strategy in preventing renal IRI. Other studies not investigating on sulforaphane also reported on the importance of Nrf2 in renal IRI. Saito et al, demonstrating an increase of Nrf2 expression in renal nuclear fractions in human kidney cells after adding indoxyl sulfate to the medium [237], a known stressor to induce oxidative stress in the kidney [238]. 

### 10.2. Liver

To the best of our knowledge, no clinical study has yet examined IRI-protective effects of sulforaphane in the context of liver transplantation. However, a study in 2019 investigated the role of Nrf2-related transcription products (CBR1) in hepatic IRI in humans. In the course of ischemia, levels of CBR1 and Nrf2 expression were enhanced. These effects were further intensified by the application of sulforaphane resulting in ~20-fold increased levels of CBR1 mRNA as compared to control specimens along with increased expression of Nrf2. Similar to the situation observed for renal IRI, various preclinical studies addressing the beneficial effects of sulforaphane in the course of hepatic IRI have been performed. These studies reported about improved organ function reflected by a decrease of liver enzymes [239,240] as well as decreased tissue injury observed at histology [239,240,241]. Sulforaphane treatment has been reported to mediate anti-inflammatory effects as its application decreased the expression of proinflammatory cytokines [239]. Along with anti-inflammatory effects, anti-oxidative effects have been observed as well involving the increase of total antioxidant capacity [241], decrease of total antioxidant status [241] and beneficial effects on the endogenous antioxidative enzymes [240]. These effects translated into a decrease of oxidative stress markers upon administration of sulforaphane. [240] Beside affecting inflammatory status and oxidative stress, resveratrol has been shown to inhibit IRI-induced apoptosis and reduce mitochondrial Na^+^/K^+^-ATPase and Ca^2+^-ATPase in liver tissue [240]. Interestingly, sulforaphane has also been able to reverse the detrimental consequences of remote IRI as reduced liver enzymes and enhanced antioxidative status has been observed [242].

### 10.3. Heart and Vessels

In the clinical setting, sulforaphane does not appear to have been tested with regard to cardiac IRI prevention. Nevertheless, the isothiocyanate has been extensively reviewed and tested in vivo, with mostly positive results.

The administration of sulforaphane was reported to improve cardiac health. This has been observed at decreased levels of enzymes characteristic for heart injury [243] or decreased luminal narrowing [244] of coronary arteries. Finally, these beneficial effects translated into preserved graft function [243,244] and superior graft survival. Interestingly, knock out mice of Nrf2, a known target of sulforaphane showed the worst graft survival [245]. This further indicates an important role of sulforaphane during IRI-induced cardiac damage. The protective effects on heart health went along with decreased apoptosis [243,244]. In addition to its role in cardiac protection, beneficial effects on liver health indicated by reduced liver enzymes have been observed. [243] Besides isolated sulforaphane, beneficial effects of steamed versus cooked broccoli against IRI-induced cardiac damage have been investigated. The authors reported about decreased post-ischemic myocardial infarction in addition to attenuated cardiomyocyte apoptosis. The effects of steamed broccoli were superior to those of cooked one. [246] Surprisingly, one study reported about contradicting results. Although this study observed amelioration of oxidative stress upon treatment with sulforaphane, no effect on improved mechanistic functions or antioxidative enzymes could be observed [247].

Effects of sulforaphane by means of ischemia reperfusion models have additionally been carried out by Silva-Palacios et al. [248] and Li et al. [249] highlighting the extensive effects of sulforaphane in cardiac injury protection in terms of SIRT 1 expression and regulation of the Nrf2 pathway, respectively.

### 10.4. Concluding Remarks

To date, sulforaphane has not been investigated in clinical trials in either of the presented organs. Nevertheless, robust evidence on its protective potential comes from preclinical studies and in vitro data. In some of these studies, a reversal of its effect in knockout mice point towards the ability of sulforaphane to induce the central oxidative stress regulator Nrf2. The latter seems to bear a crucial role during SOT, as Nrf2 -/- mice showed reduced support of cardiac grafts and human kidney cell demonstrated the upregulation of Nrf2 upon induction of oxidative stress. A potential role of Nrf2 was even observed in clinics, where hepatic IRI lead to the upregulation of Nrf2, which was further increased by administration of sulforaphane. In addition to targeting Nrf2, some studies observed the activation of SIRT1 by sulforaphane. The activation of crucial mediators of cell survival together with very promising in vivo data uncovering its valuable multifaceted protective effects encourages exploring the full potential of this compound against IRI during SOT in future clinical trials.

## 11. Vitamin C

Vitamin C is a water-soluble vitamin that mainly occurs in fruits and vegetables [91]. Despite acting in a hydrophilic environment, vitamin C can also impact hydrophobic compartments as it acts synergistically with lipophilic vitamin E [250]. Vitamin C possesses direct as well as indirect antioxidative effects. The direct effects are mediated by scavenging of free radicals, thereby neutralizing them [91]. Indirect antioxidative properties arise from stabilizing tetrahydrobiopterin (BH_4_), which is a cofactor of eNOS. Its stabilization prevents eNOS-uncoupling and subsequent superoxide generation. Besides preventing eNOS uncoupling, vitamin C also inhibits NAPDH oxidase, which is a crucial source of ROS [17]. In addition to its antioxidative effects, vitamin C provides anti-inflammatory effects by inhibiting the secretory phospholipase A_2_, the latter being a source of proinflammatory mediators [17]. Based on these mechanisms, vitamin C has been shown to enhance microcirculation [251,252], reduce endothelial permeability and reduce inflammation [251]. For this reason, vitamin C appears as an attractive candidate to ameliorate the negative consequences of IRI-induced injuries. However, it should be noticed that oral doses from 400 mg daily are capable of saturating the system and excess vitamin C would be eliminated via the urinary route. Consequently, oral vitamin C intake can only yield plasma concentrations up to 80 µmol/l. As these concentrations are not sufficient for direct free radical scavenging, intravenous application should be preferred [17]. 

### 11.1. Kidney

The effect of vitamin C on IRI in the course of kidney transplantation was only investigated in a very limited number of patients in two clinical studies. Hower et al. [253] tested the effect of 500 mg intraoperatively administered vitamin C, prior to reperfusion in 17 patients. Reperfusion led to local increases of MDA as oxidative stress marker and IL-6 as marker of inflammation in renal blood samples. The treatment group showed a non-significant trend towards amelioration of inflammation and oxidative stress. In a second study by Norio et al. [250] renal cadaveric grafts were treated with 500 mg vitamin C prior to implantation. The authors observed similar results, as the treatment neither affected the incidence on delayed graft function, the rejection rate nor the graft function per se. However, a trend towards shorter duration of delayed graft function could be observed. 

In contrast to the clinical studies, preclinical studies showed promising results reflected by amelioration of oxidative stress and inflammation [254] as well as restoration of the endogenous antioxidant capacity [255] and organ function [256] in mouse models of IRI. However, it should be mentioned that the trials involved only a very limited number of patients and the utilized vitamin C quantities were rather low. In order to be able to estimate the true potential of vitamin C, larger trials administering higher amounts should be considered.

### 11.2. Liver

Clinical studies examining the effect of vitamin C on the prevention of IRI in liver are scarce. There are clinical trials in which vitamin C was used as part of an antioxidant mix [257,258,259] but these do not allow any causal statement to be made about vitamin C as a single substrate. In a South Korean study, blood samples of recipients of living donor liver grafts were investigated. The authors found vitamin C to be supportive for prevention of hyperfibrinolysis by improving clot rigidity [260]. In contrast, numerous animal studies addressing the prevention and/or reduction of IRI by the use of vitamin C are availablein respect to the local hepatic damage, as well as remote organ injury caused by liver reperfusion. While vitamin C seems to improve classic liver biomarkers like ALT and AST, it also seems to have a positive impact on mitochondrial health as assessed through glutamate dehydrogenase activity after ischemia reperfusion. Overall, there seems to be a broad consensus in the in vivo associated literature that hepatic ROS as a consequence of IRI can represent a therapy-limiting problem and vitamin C, as a potent radical scavenger, could potentially counteract. [261,262,263]. Kitamura et al. [264] ultimately justified the use of antioxidants in the context of IRI as they showed that hepatic vitamin C levels are more than halved as a result of ischemia reperfusion.

### 11.3. Heart and Vessels

To the best of our knowledge, no clinical studies on the beneficial effects of vitamin C in the course of IRI in heart transplantation have been conducted. However, a decrease of the vitamin C levels after coronary interventions in conjunction with increasing levels of oxidative stress have been observed. These levels remained low even days after surgery. [252] Not surprisingly, treatment with vitamin C could attenuate the oxidative burden and increase the total antioxidant capacity in various clinical settings of myocardial ischemia-reperfusion injury [265,266,267]. Similar effects have also been reported in combination with vitamin E [268]. Besides its antioxidative effects, vitamin C treatment protected the cardiac tissue against perioperative injuries in the setting of percutaneous coronary intervention [269,270]. These beneficial effects were accompanied by protection against endothelial injury in the clinical [271] and ex vivo [272] setting. The cardiac protection translated into attenuation of postoperative arterial fibrillation (AF) in various clinical studies, which were analyzed by several meta-analyses [273,274,275]. In one clinical study, vitamin C treatment could increase the beneficial effects of ß-blocker against AF in patients undergoing CABG [276]. However, some clinical studies were not able to find significant effects on heart enzymes [267,277], inflammation markers [278] or AF [266]. In animal models, beneficial effects of vitamin C supplemented preservation solution in terms of graft protection have been observed [279].

### 11.4. Concluding Remarks

Although vitamin C is considered a classical antioxidant being appreciated for a long time already for various indications, the number of studies conducted strongly depends on the respective organ. Whereas hardly any literature for kidney and liver exists in terms of clinical studies, the use of vitamin C against coronary-intervention-induced IRI has been extensively researched. Interestingly, clinical studies addressing the kidneys could not find beneficial effects whereas preclinical studies in addition to the majority of combined clinical and preclinical studies in the liver and heart proved vitamin C to be effective in preventing IRI. At this point it should be mentioned, that these studies utilized rather low concentrations of vitamin C (500 mg). Observational studies pointed towards a specific role of vitamin C in hepatic and cardiac IRI, as they observed vitamin C depletion after IR insults. If administration of vitamin C therefore serves to replete the pool rather than having additive effects remains to be investigated. The same applies for the question, if vitamin C depletion after IR also occurs in renal tissue. If this was not the case, it might explain the contradicting clinical results. Vitamin C is characterized by good tolerance, low toxicity and inexpensive availability. These attractive properties should be used to conduct future clinical trials in order to answer remaining questions and discover the full potential of this micronutrient.

## 12. Conclusions

Despite emerging techniques in the field of SOT, IRI-induced complications still present a therapy-limiting issue. Since oxidative stress plays an important role in the development of IRI, compounds bearing antioxidant properties appear as attractive candidates. For this reason, a variety of different micronutrients and natural compounds has been tested for their protective effects against IRI in the context of SOT. 

Yet, most of the clinical study results are preliminary, and some failed to translate the beneficial effects from animal studies into the clinical setting. Additionally, a high degree of heterogeneity between studies exists in terms of route of application, treatment of donor or recipient vs. graft flushing or the use of cadaveric grafts. Furthermore, different clinical endpoints were selected ranging from very basal conditions, like the degree of oxidative stress and inflammation markers to clinical consequences like the duration of hospital stays or the graft function per se. 

However, in various preclinical studies beneficial effects of selected micronutrients and natural compounds have been shown. Beside their well-known antioxidant properties, pleiotropic effects like antiapoptotic, anti-inflammatory or antiarrhythmic effects have been observed translating into remarkable protection of grafts and improved recovery of affected animals. Due to these beneficial effects, along with their easy availability and their safety profile, as well as low cost, the application in the course of SOT should be taken into consideration. In this respect, further clinical trials focusing on optimization of micronutrients/natural compound treatment regimens are required. These should consider findings from preclinical studies and, thus, especially focus on optimization of the treatment duration, dosing and route of application. 

Finally, although micronutrients and natural compounds appear to be attractive candidates especially in terms of beneficial effects on ECD grafts due to their unique properties, very little research has focused on this certain topic so far. For this reason, future studies should especially focus on modulating ECD survival. 

**Table 1:** Summary of the main clinical findings of the respective micronutrients with reference to the individual organs. Main categories of outcomes are presented in bold.

MN	Organ	Application	Route of Administration	Dosage	Outcome
**ALA**	**Kidney**		*i.v.* [21]	600 mg once immediately before surgery [21]	**anti-inflammatory effects** [21]
					downregulation of proinflammatory cytokines (IL-8, IL-6) [21] reduced clinical pancreatitis [21]
		kidney/ pancreas transplantation [21]			**anti-oxidative effects** [21]
					upregulation of HO-1 [21]
	
					endothelium protective activity (eNOS increased, ET-1 decreased) [21]
	**Liver**	Transplantation [27] Resection [28]	direct injection [27] *i.v.* [28]	600 mg once immediately before ischemia and once 15 min before reperfusion [27] 600 mg 15 min before ischemia [28]	**improved liver function**
					decreased bilirubin levels (in ECD graft subgroup) [27,28]
					reduced frequency of post-perfusion syndrome [27]
					trend towards reduced no. of rejections [27]
					preserved liver function (reduced levels of ALT and AST) [28]

					**anti-apoptotic effects** [28]

					**energy metabolism** [28]
					preservation of hepatic ATP-content [28]
	**Heart**	CPB [35]	Priming solution [35]	200 mg [35]	**anti-inflammatory effects** decreased proinflammatory cytokines (IL-6, IL-8) [35] decreased levels of complement factors C3 and C4 [35] decreased levels of CRP [35]
**Astaxanthin**	**Kidney**	Transplantation [51]	*p.o.* [51]	3 × 4 mg capsules for 12 months [51]	**no effect on:** arterial stiffness [51] oxidative stress [51] inflammation [51]
	**Liver**	-	-		-
	**Heart**	-	-		-
**CoQ10**	**Kidney**	-	-		-
	**Liver**	-	-		-
	**Heart**	CABG [81,82,83,84] CAD [82]	*p.o.* [82,83] nm [81] *i.v.* [84] ex vivo [78]	3 × 50 mg/day for 7 days [81] 1 × 50 mg/day for 7 days [82] 3 × 50 mg/day (< 60 kg) for 7–10 days [83] 3 × 60 mg/day (> 60 kg) for 7–10 days [83] 5 mg/kg/2h [84] 400 µM [78]	**anti-inflammatory effects** reduced levels of conjugated dienes [81] reduced levels of MDA [81] **organ function** reduced levels of CK [81] less incidences of ventricular arrhythmias [81,83] reduced requirement of total inotropic [83] reduced mediastinal drainage [83] reduced blood requirement [83] decrease of myocardial cellular injury [84] prevention of left ventricular depression [84] no effect on infarct biomarkers CK-MB and troponin [82] decreased duration of hospital stays [83], no effect in [82] attenuated decline of contractile function [78]
**Curcumin**	**Kidney**	Transplantation [94,95]	*p.o.* [94,95]	400 mg curcumin, 100 mg quercetin, 10 mg bromelain/day [94] 480 mg curcumin, 20 mg quercetin for 1 month starting within 24 h after transplantation [95]	**organ function** increased urine output [94] improved creatinine clearance [94,95] early graft function [95] fewer acute rejection events [95] **anti-oxidative effectsincreased HO-1 expression** [95] decreased level of iso-prostaglandins [94]
				
	**Liver**	-	-		-
				
				
	**Heart**	CABG [127]	*p.o.* [127]	4 × 250 mg/day 3 days before surgery and 5 days after [127]	**organ function** less postoperative myocardial infarctions [127] **anti-inflammatory effects** reduced CRP levels [127] **anti-oxidative effects** reduced levels of MDA [127]
**Glycine**	**Kidney**	-	-		-
	**Liver**	Transplantation [150,151,152,154]	*i.v.* [150,151,154] hepatic arterial and portal vein flush [152]	250 mL 300 mM 1hr before reperfusion + 25 mL 300 mM for 1 week [150,151] 250 mL 4.4 % during anhepatic phase and once daily for 1 week [154] 2 mM [152]	**organ function** reduced incidence of primary graft dysfunction [150] reduced levels of liver enzymes AST and ALT [150,151,152] reduced occurrence of ischemic type bile duct strictures [152] reduced IRI in subgroup of high levels of glycine [154] no effect on patient or graft survival nor on liver enzymes [154]
	**Heart**	-	-		-
**n-3 PUFA**	**Kidney**	Transplantation [160,161]	*p.o.* [160,161]	1.2 - 5.4 g/day (metaanalysis) [160] 0.6–5,4 g/day < 12 months (metaanalysis) [161]	**organ function** trend towards improved renal function (no significant effects) [160,161] trend towards improved recovery after rejection (no significant effects) [160,161] trend towards improved filtration fraction (no significant effects) [160,161]
	**Liver**	Transplantation [170,171,172,174,175] liver resection [157,176] donor liver recovery [173] open liver surgery [176]	*p.o.* [174] parenteral nutrition [157,170,171,172,173,176] enteral nutrition [170,174,175]	100 mL/day (*i.v.*), 600 mL/day (*p.o.*), 2 mL/kg/day (*i.v.*) [170] 2 mL/kg 10% for 7 days from second day after surgery [171,172] 2 g/day before surgery and for at least 5 posttransplanation days [174] 2 g/day for at least 5 posttransplant days [175] 10 g fish oil the evening before the surgery and before anesthesia [176] 100 mL/day 10% for 5 days after surgery [157] 7 mL/kg 20 % fish oil once before surgery and once on postoperative day 0 [173]	**organ function** decreased liver enzymes (AST [170,173] (no effect [171]), ALT [171,172,173]) and bilirubin [157] (not for [170,171]) dampened hepatic cell injury [171,172] no effect on mortality or rejection episode [170,174,175,176] shorter duration of hospital stays [157,171] no impact on ICU duration [174,175] increased regeneration index [173] increased levels of hepatic growth factor [173] increased donor liver volume [173] no effect on inflow occlusion [176] **anti-inflammatory effects** reduced portal inflammation [171,172] decrease of WBC [157,173] reduced levels of CRP [173]
	**Heart**	CABG [159] CABG or valve surgery [182]	parenteral nutrition [159] *p.o.* [182]	100 mL infusion (1.25–2.82 g EPA/1.44–3.09 g DHA) was given one day before surgery and repeated 4 h before surgery [159] 2 g/day for 7 days before surgery [182]	**organ function** faster return to aerobic metabolism [159] faster clearance of lactate [159] fast onset of O_2_ consumption [159] lower occurrence of arterial fibrillation [182] **anti-oxidative effects** reduced levels of biomarkers of oxidative stress [182] inhibition of prooxidative enzymes [182] increased activities of endogenous antioxidants [182] **anti-inflammatory effects** reduced levels of biomarkers of inflammation [182]
**NAC**	**Kidney**	transplantation [186,187,188,189,190,194] CABG [191,192,193]	*p.o.* [186,187,188,190,193] treatment of donors [189] *i.v.* [191,192,194]	600 mg twice a day for 8 months [186] 600 mg twice a day for 7 postoperative days [187] 600 mg NAC, 6 hours before grafting and 12 and 18 hours after transplantation [189] 600 mg 3-times a day 6 hours before grafting and 12 + 18 h after transplantation [190] 2 intraoperative and 2 postoperative doses of 600 mg NAC i.v. within 24 h [191] 600 mg 2 h before surgery, 5 days postoperatively 2 × 600 mg/day [188] 150 mg/kg *i.v.* bolus before the surgery and 150 mg/kg/day for 5 postoperative days [192] 2 × 600 mg 24 h before the surgery + 2 postoperative days [193] 5 g in 5% glucose solution [194]	**organ function** reduced serum creatinine (no effect [189]),186,187], improved Cockroft-Gault formula [186] improved eGFR [187] fewer events of delayed graft function (no effect [189]) [187,188] trend towards delayed graft function (reduced NGAL and IL-18) [190] improved graft function (reduced RGF rate and p-NGAL levels) [188] trends towards serious adverse events, duration of hospital stays, hospital mortality [191] no effect on acute kidney injury [192,193] reduced homocysteine levels [194] **anti-oxidative effects** lower levels of thiobarbituric acid reactive substance [187] no effect on markers of oxidative stress [186]
	**Liver**	transplantation [197,199] hepatic resection [198]	*i.v.* [197,198] perfusion solution [199]	15 min of 30 mg/kg *i.v.* one hour before liver harvesting and 150 mg/kg estimated liver weight into portal vein before cross clamping [197] 10 g/24 h in 250 mL at time of transection + 3 days postoperatively or until a fall in post-operative ALT [198] 2 g in 1 l UW solution for perfusion [199]	**organ function** improved graft survival [197] no effect on hepatic biomarkers (ALT, AST, bilirubin) [197,198] higher frequency of post-hepatectomy liver failure (grade A) [198] trend towards lower frequency of post-hepatectomy liver failure (grade B&C) [198] trend towards reduced overall complications [198] no effect on duration of hospital stays [199] no effect on vascular complications [199] no effect on hepatic biomarkers (ALT, AST, bilirubin) [199] no effect on inotrope requirements [199]
	**Heart**	cardiac surgeries [200] transplantation [201]	*p.o.* [200,201] *i.v.* [200] cardioplegia [200] *p.o.* + *i.v.* [200]	10–460 mg/kg [200] 500 mg/day for 10 weeks [201]	**organ function** trend towards reduced mortality [200] trend towards reduced acute cardiac insufficiency [200] reduced length of hospital stays [200] no effect on plasma homocysteine levels [201] no effect on brachial artery endothelial function [201]
**Resveratrol**	**Kidney**	-	-		-
	**Liver**	-	-		-
	**Heart**	-	-		-
**Sulforaphane**	**Kidney**	-	-		-
	**Liver**	-	-		-
	**Heart**	-	-		-
**Vitamin C**	**Kidney**	Transplantation [250,253]	Intraoperatively [253] treatment of grafts [250]	0.5 mg/mL in 300 mL perfusion solution [250] 500 mg intraoperatively prior to reperfusion [253]	**anti-inflammatory effects (not statistically significant)** [253] **anti-oxidative effects (not statistically significant)** [253] **graft function** no effect on delayed graft function [250] no effect on rejection rate [250] no effect on graft function [250] trend towards shorter duration of delayed graft function [250]
				
	**Liver**	Transplantation [260]	-	Varying concentrations AA [260]	**blood circulation** improved hyperfibrinolysis by improvement of clot rigidity [260]
				
	**Heart**	CABG [265,266,274,275,276,277,278] percutaneous coronary intervention [269,270] cardiac surgery [267,273] PAD [271] valve replacement surgery [274, 278]	pump circulation during surgery [265] *i.v.* [266,267,270,271,273,274,277] *p.o.* [273,274,276,278] cardioplegic solution [277]	25 mg/kg through cardiopulmonary bypass during surgery [265] 500 mg *i.v.* intra-operatively 30 minutes before reperfusion and then post-operatively, 120 minutes after reperfusion [266] *i.v.* after induction, just before aortic de-clamping [267] varying (metaanalysis) [269,273,274,275] 2 g orally for at least 3 days before surgery [276] 3-g vitamin C infusion within 6 hours percutaneous coronary intervention [270] 24 mg/min intra-arterial *(i.a.)* during reperfusion [271] 5 g of *i.v.* before anesthesia and 5 g in cardioplegic solution [277] 2 g orally the night before surgery. From the day of surgery through postoperative day 4, 500 mg every 12 hours orally [278]	**antioxidative effects** decrease of oxidant burden [265,266,267] increase of antioxidant capacity [265,266,267] **tissue integrity** protection against perioperative injuries [269,270] protection of endothelial lining [271] no effect on heart enzymes [267,277] **anti-arrhythmic effects** less episodes of arterial fibrillation [273,274,275] synergistic effects with ß-blocker against AF [276], no effect [266] **anti-inflammatory effectsno effects** on inflammation markers [278]

**Table 2:** Summary of the main preclinical findings of the respective micronutrients with reference to the individual organs. Main categories of outcomes are presented in bold.

Micronutrient	Organ	Species	Route of administration	Outcome
**ALA**	**Kidney**	rat [23,24,25,26]	*i.p.* [23,24,25,26]	**organ function** [24] decreased expression of aquaporins and sodium transporters [23,26] preserved kidney function (decreased creatin and urea levels) [23,26] **anti-inflammatory effects** [23] **antioxidative effects** [25] endothelium protective activity (eNOS increased, ET-1 decreased) [24] synergistic effects with XO inhibitor febuxostat [25]
	**Liver**	rat [29,30,31,33,34]	*i.v.* [29,31] *i.p.* [30,33,34]	**preservation of tissue integrity** reduced levels of LDH [29], ALT [30,31], AST [30] increased ATP content [30] increased survival of animals [33,34] **anti-apoptotic effects** downregulation of proapoptotic proteins [33,34] **anti-inflammatory effects** inhibition of NFκB [29,31] reduced secretion of proinflammatory cytokines [30] **anti-oxidative effects** increased endogenous antioxidants [29,31] reduced activity of prooxidant enzymes [30] effects were mainly mediated via PI3K/Akt axis [29]
	**Heart**	rat [36,37,38,39,40,41] pig [42]	*p.o* [36,40,42] *i.v.* [37] *i.p.* [38] ex vivo [39,41]	**enhanced graft survival** [36] reduced LDH and CK release [37,38] decreased infarct size [37] **anti-oxidative and anti-toxicity effect** induction of ALDH_2_ expression (detoxification of aldehydes) [39] **preserved cardiac function** [40] decreased number of arrythmias [41] improved coronary flow [40] increased peak arterial pressure [40] **anti-inflammatory** inhibition of NFκB and ERK [42] **anti-arrhythmic effects** activation of K-dependent ATPase by release of H_2_S [41] activation of PI3K/Akt/Nrf2/HO-1 pathway [37]
**Astaxanthin**	**Kidney**	in vitro [52] mouse [52] rats [53]	*p.o.* [52,53]	**anti-oxidative effects** [52] protection against H_2_O_2_-induced cell death [52] increased SOD [52,53] decreased MDA [52,53] decreased total thiols and 8-hydroxydeoxyguanoside [53] **graft function** improved serum levels of urea nitrogen and creatinine [52] improved histopathologic scores [52] **anti-apoptotic effects** decrease of caspase (3, 8, 9) [53] **anti-inflammatory effects** decreased levels of TNF-a and IL-6 [53]
	**Liver**	rats [54] mouse [55,56]	intragastric intubation [54] *p.o.* [55,56]	**antioxidative effects** reduced levels of xanthine oxidase [54] reduced tissue protein carbonyl levels [54] reduced level of oxidative stress [55] **tissue preservation** reduction of histopathologic scoring [54] **anti-apoptotic** [55,56] induction of autophagy [56] decreased level of necrosis [55] **anti-inflammatory effects** decreased level of macrophage infiltration [55]
	**Heart**	rabbit [58] mouse [59] in vitro [60]	*i.v.* [58] *p.o.* [59]	**graft function** reduced infarct size [58,59] reduced inflammation and myocardial injury [58] **anti-inflammatory effects** inhibition of complement activation [58] **antioxidative effects** improved mitochondrial parameters [59] cell protection [60]
**CoQ10**	**Kidney**	rat [62,63,64,65]	*i.m.* [62] *p.o.* [65] *i.p.* [64]	**improved organ function** increased survival rate [62] improved kidney function [63] improved histopathological scores [64] improved renal morphology [65] decreased blood urea nitrogen [65] enhanced rate of ATP resynthesis [62] **anti-oxidative effects** [64] decreased NO-levels [65] decreased MDA levels [65] improved SOD activity [65] **anti-inflammatory effects** decreased levels of TNF-α [65]
	**Liver**	rat [66,67,69,70,71,72] mouse [68]	*i.v.* [66,68,71] *i.p.* [70] *p.o.* [67,69]	**improved organ function** decreased activities of serum glutamic oxaloacetic transaminase [66] decreased activity of serum glutamic pyruvic transaminase [66] increased survival rate [66,72] increased level of total protein [66] improved histopathology [67] reduced serum ALT levels [68] **anti-oxidative effects** [67] increased levels of glutathione [69,71] decreased MDA levels [69] protection of endogenous CoQ10 levels [70,71] improved mitochondrial function [70] **anti-apoptotic effects** reduced biomarkers of apoptosis [67]
	**Heart**	rat [85,86,87,90] pig [88] rabbit [89]	*i.m.* [85] *i.p.* [85] *i.v.* [86,87,90] *p.o.* [88] intracardial injection [89]	**improved energy metabolism** increase of aerobic energy production [85] preservation/delay of aerobic efficiency [85] preservation of mitochondrial function [90] **improved organ function** [87] improved myocardial function [86] decreased oxidative injury [86] reduced cardiac stunning time [88] reduced area of infarction risk [89]
**Curcumin**	**Kidney**	rats [96] mouse [96]	*i.p.* [96] *i.v.* [96] *p.o*. [96]	**anti-inflammatory effects** [96] inhibition of NFκB [96] inhibition of NMDA [96] induction of Nrf2 pathway [96] **anti-apoptotic effects** [96] inhibition of caspase 3 [96] induction of NO/cGMP/PKG pathway [96] **antifibrotic effects** [96] activation of APPL [96] inhibition of Akt [96]
	**Liver**	mouse [108] rat [109,110,111,112,113,114,116] in vitro [114,115]	*i.p.* [108] *p.o.* [109,110] *i.v.* [111,113] preservation solution [116]	**prevention of liver injury** [108,109,110,111] attenuated liver enzymes AST, ALT [112,113] restoration of ATP content [110] protection against injury during warm und cold storage [116] increased survival [116] **anti-inflammatory effects** reduced expression of proinflammatory cytokines [114] reduced expression of adhesion molecules [113,115] anti-inflammatory priming of Kupffer cells [108] inhibition of NFκB [114] lower pulmonary levels of TNFα and MMP-9 activity [112] **antioxidative effects** activation of antioxidant enzymes [112] upregulation of HO-1 [111] Hsp70 expression [111]
	**Heart**	rat [128,129,130,131,135,280] in vitro [129,134,137,138] mouse [132,133]	intracardial injection [128] perfusion solution [129,280] *p.o.* [130,132,133,135] *i.p.* [131]	**improved organ function** restoration of organ function [128,129,130] attenuation of cardiac lesions [131,132,133] amelioration of cold-storage induced damage [134] **anti-apoptotic effects** [128,132,135] **anti-inflammatory effects** [132] **anti-oxidative effects** [132,137,138] **mechanisms** activation of JAK2/STAT3 pathway [128,133,280] downregulation of Notch1 pathway [138]
**Glycine**	**Kidney**	ex vivo [141,142] rat [140,144,145] in vitro [143]	preservation solution [140,141,142] *i.p.* [145] *p.o.* [144]	**improved organ function** reduced cellular damage (LDH release and alkaline phosphatase) [140,142,143] protection against IR injury [141] no effect on regeneration of ATP content or mitochondrial function [142] cell protection (reduced LDH release) improved glomerular filtration rate [144] reduced urinary LDH [144]
	**Liver**	rat [148,281]	*p.o.* [281] perfusion solution [148]	**improved organ function** reduced liver injury [281] **anti-inflammatory effects** inhibition of Kupffer cell activation [148]
	**Heart**	rat [155] pig [156]	*p.o.* [155] *i.v.* [156]	**improved organ function** decreased intimal and medial thickening [155] inhibition of proliferation and migration of VSMC [155] increased volume load [156] increased postischemic right ventricular end-diastolic compliance [156] no effect on maximal right ventricular developed pressure [156] **anti-inflammatory effects** reduced infiltration of macrophages [155]
**n3-PUFA**	**Kidney**	mouse [162] rat [163,164,165]	*p.o.* [162,163,164,165]	**improved organ function** preserved tubular integrity [162] less histologic damage [163] improved renal function [165] **anti-oxidative effects** [163,164] **anti-apoptotic effects** [164]
	**Liver**	rat [177,180] mouse [179] in vitro [181]	*p.o.* [177,180] *i.v.* [179]	**improved organ function** hepatic tissue protection [177,179,180] **anti-inflammatory effects** [180,181] inhibition of NFκB by activation of PPARα [180] inhibition of NFκB by activation of PI3K/Akt [181] inhibition of TLR4 recruitment [177]
	**Heart**	mouse [183,184] rat [185]	*i.p.* [183,184] *p.o.* [185]	**improved organ function** increased graft survival [183,184] **anti-inflammatory effects** shift towards Treg via activation of PPARγ [183,184] inhibition of macrophage recruitment [185] inhibition of MCP-1/IP-10 [185]
**NAC**	**Kidney**	-	-	-
	**Liver**	-	-	-
	**Heart**	rat [202]	*i.v.* [202]	**improved organ function** improved shortening of isograft [202] decreased serum LDH [202] improved liver biomarkers (AST, ALT) [202] **anti-inflammatory effects** decreased serum TNFα [202] decreased serum IL-1 [202] **anti-oxidative stress** increased SOD activity [202] increased SOD/LPO [202]
**Quercetin**	**Kidney**	rat [91,98,99,100,101] pig [97] in vitro [102] mouse [103]	*i.p.* [91,98,99,100,101,103] preservation solution [97]	**anti-oxidative effects** [91,97,98,99] decrease of TBARS [100] decreased MDA [101] increased level of antioxidant enzymes [101] **anti-inflammatory effects** [91,99] **anti-apoptotic effects** [99,102] **induction of autophagy** [103]
	**Liver**	rat [98,104,105,107] mouse [106]	*i.p.* [98,104] *p.o.* [106] *i.m.* [107]	**improved organ function** improved level of liver enzymes AST, ALT [98,104] attenuation of liver injury [98] **anti-oxidative effects** [104,105] **anti-apoptotic and anti-autophagy effects** [106] downregulation of ERK/NFκB [98] synergistic effects with remote ischemic reperfusion preconditioning [98] synergistic effects with Fe^2+^ chelator desferrioxamine [107]
	**Heart**	rat [117,120,121,122,123,124,282] in vitro [118,119,121,283] ex vivo [118] mouse [119]	preservation solution [117,118] *i.p.* [119] *p.o.* [119,120,123,124,282]	**improved organ function** improved cardiac flow [117] alleviated cardiac dysfunction [118] decreased infarct size [119,121,122] **anti-oxidative effects** improved mitochondrial function [124] **mechanisms** activation of JAK2/STAT3 pathway [282] activation of PI3K/Akt pathway [118] inhibition of HMGB1 pathway [121] upregulation of PKCε [283] **anti-apoptotic effects** inhibition of SIRT1/PGCα signaling [123] **anti-inflammatory effects** inhibition of NFκB [119] activation of PPARγ [119]
**Resveratrol**	**Kidney**	pig [210] rat [211,212,213,214,215,216,284,285] in vitro [216]	preservation solution [210] *i.p.* [213,216,285] *i.v.* [211,215] *p.o.* [212,214,284]	**improved organ function** [212,214] improved glomerular filtration [210] improved proximal tubular function [210] slowed chronic loss of function [210] slowed chronic onset of histological lesions [210] reduced mortality [211] attenuation of increased serum urea [213] reduced creatinine levels [211] **improved organ morphology** [210,211,214] attenuated interstitial fibrosis [210] attenuated tubular atrophy [210] preservation of glomerular number reduction [213] preservation of glomerular collapse [215] **anti-oxidative effects** [210,212,216] restoration of antioxidant enzyme pool [214] decreased levels of MDA [284] decreased levels of TBARS [214] decreased levels of lipid peroxidation products [211] increased levels of GSH [284,285] increased levels of NO [211,214,285] **anti-apoptotic effects** [210] Nrf2/TLR4/caspase 3 axis [212] induction of autophagy via SIRT-1 [216] **anti-inflammatory effects** [212]
	**Liver**	rat [219,220,221,222,223,224,225] mouse [218]	preservation solution [223] reperfusion solution [223] *i.p.* [218,222] *i.v.* [219,220,221,224,225]	**improved organ function** decreased plasma aminotransferase activity [219] reduced levels of histological liver injury [219] improved bile production [220] improved portal vein flow volume [220] preserved sinusoidal endothelial cells [220] ameliorated ATP levels [220] decreased VEGF [224] **anti-oxidative effects** inhibition of lipid peroxidation [223] protection of mitochondria [223] reduced levels of MDA [219] increased levels of GPx [219] increased levels of CAT [219] expression of Txnip [221] reduced expression of thioredoxin [221] attenuation of GSH depletion [225] increased GSH reductase [225] increased SOD [219,225] attenuation of HIF-1α [224] **anti-inflammatory effects** decreased levels of TNFα [218,220] suppressed leucocyte infiltration [218] decreased levels of IFNγ [218] increases apoptosis of anti-graft lymphocytes [222] **anti-apoptotic effects** reduction of apoptotic and necrotic markers [223] reduced release of cytochrome C [223]
	**Heart**	rat [227,228,229,230,231,232,233]	perfusion solution [228,233] *i.v.* [229,230,232] *i.p.* [231] *p.o.* [227]	**improved organ function** [228,232] reduced rhythm disturbances [231] **anti-apoptotic effects** [227,229] reduced infarct size [227,228,229,230,231] reduced levels of LDH [230,231,232] reduced levels of CK [231,232] alleviated myocardial injury [233] **anti-oxidative effects** increased expression of Nrf2 [232] increased expression of HO-1 [232,233] enhanced activity of SOD and GPx [232] reduced levels of superoxide [227,232] reduced levels of MDA [232] suppression of myeloperoxidase [229] **anti-inflammatory effects** attenuation of TLR4 and NFκB expression [229] reduced activation of iNOS [231] reduced generation of TNFα [230] **improved endothelial function** increased VEGF-B [227] increased p-eNOS [226,227,230] increased NO-production [226]
**Sulforaphane**	**Kidney**	in vitro [234] rat [234,235,236]	*i.v.* [234,235,236]	**improved organ function** reduced cellular toxicity [234] induction of phase II enzymes [234] reduced renal injury [234,236] improved histopathologic scoring [234] lower blood urea nitrogen levels [236] decreased serum creatinine levels [236] improved kidney function [235] **anti-oxidative effects** inhibition of lipid peroxidation products [234] preservation of mitochondrial microstructure [236] increase of SOD-2 expression [236] **anti-inflammatory effects** [235] **anti-apoptotic effects** [235]
	**Liver**	mouse [239] rat [240,241,242]	*i.p.* [240,241,242] *p.o.* [241]	**improved organ function** decreased levels of liver enzymes (AST and ALT) [239,240,242] decreased tissue injury [239,240,241] **anti-inflammatory effects** decreased levels of TNFα [239] decreased levels of IL-6 [239] **anti-oxidative effects** trend towards increased Nrf2-related transcription products [239] improved total antioxidant capacity [241] improved total antioxidant status [241] decreased levels of MDA [240] decreased levels of MPO [240] improved activity of SOD [240,242] improved levels of GSH [240,242] improved activity of GPx [240,242] increased expression of Nqo1 [240] increased expression of Nrf2 [240] increased expression of HO-1 [240] induction of Nrf2 pathway [242] **anti-apoptotic effects** [240]
	**Heart**	rat [243,246,247,249] mouse [244]	*p.o.* [246] *i.p.* [244,245,247] *i.v.* [243] intracardial injection [248,249]	**improved organ function** less post-ischemic myocardial infarction [246] decreased cardiomyocyte apoptosis [246] expression of phase II enzymes [246] increased graft survival [245] decreased levels of TnT, CK, CK-MB and LDH [243] improved liver function (decreased levels of AST and ALT) [243] improved graft function [243,244] improved survival [243] **anti-apoptotic effects** decreased markers of apoptosis (caspase 3) [243,244] increased expression of SIRT1 [248,249] **antioxidative effects** decreased oxidative stress [247] no effect on CAT [247] no effect on GPx [247]
**Vitamin C**	**Kidney**	rat [254,256] mouse [255]	*i.v.* [254] *p.o.* [255] *i.p.* [256]	**anti-oxidative effects** [254] **anti-inflammatory effects** [254] restoration of endogenous antioxidant capacity [255] **improved graft function** [256]
	**Liver**	rat [261,263]	*i.v.* [261] *i.p.* [263]	**improved organ function** improved liver function biomarkers (ALT, AST) [261] **mitochondrial health** improved glutamate dehydrogenase activity [261] **anti-oxidative effects** [263]
	**Heart**	mouse [279]	preservation solution [279]	**graft protection** [279]

**Table 3:** List of abbreviations.

AF	artrial fibrillation
Akt	protein kinase B
AMPK	AMP-activated protein kinase
ALA	alpha lipoic acid
ALDH2	Aldehyde dehydrogenase
ALT	alanine aminotransferase
APPL	PH domain and leucine zipper 1
ARE	antioxidant response element
AST	aspartate aminotransferase
BH4	tetrahydrobiopterin
CABG	coronary artery bypass graft
CAT	catalase
CBR1	carbonyl reductase 1
cGMP	cyclic guanosine monophosphate
CK	creatine kinase
CK-MB	creatine kinase muscle-brain type
CoQ10	Coenzyme Q10
CRP	c-reactive protein
DAMP	damage associated molecular pattern
DHA	docosahexaenoic acid
ECD	extended donor criteria
eGFR	estimated glomerular filtration rate
eNOS	endothelial NO synthase
EPA	eicosapentaenoic acid
ER	endoplasmic reticulum
ERK	extracellular-signal regulated kinase
ET-1	enodthelin 1
GFR	glomerular filtration rate
GPx	glutathione peroxidase
GSH	gluthathion
HDL	high density lipoprotein
HIF-1α	hypoxia inducible factor 1 a
HMGB1	high mobility group box 1
HO-1	hemeoxygenase 1
Hsp70	heat shock protein 70
i.m.	intramuscular
iNOS	inducible NO-synthase
i.p.	intraperetoneal
i.v.	intraveneous
ICU	intensive care unit
IL-18	interleukin 18
IL-6	interleukin 6
IL-8	interleukin 8
IP-10	interferon γ-induced protein 10
IR	ischemia reperfusion
IRI	ischemia-reperfusion injury
JAK2	Janus kinase 2
Keap1	Kelch-like ECH-associated protein 1
LDH	lactate dehydrogenase
L-NAME	N γ-nitro-L-arginine methyl ester
LPO	lipid hydroperoxide
MCP-1	monocyte chemoattractant protein-1
MDA	malondialdehyde
MMP-9	matrix metalloprotase 9
MPO	myeloperoxidase
n3-PUFA	omega-3 polyunsaturated fatty acids
NAC	N-acetylcysteine
NFκB	nuclear factor "kappa-light-chain-enhancer" of activated B-cells
NGAL	neutrophil gelatinase-associated lipocalin
NMDA	N-methyl-D-aspartate receptor
Nqo1	NAD(P)H dehydrogenase [quinone] 1
Nrf2	Nuclear factor erythroid 2-related factor 2
p.o.	per orales
PGCα	Peroxisome proliferator-activated receptor-gamma coactivator-1α
PI3K	phosphoinositide 3-kinase
PKCε	protein kinase C ε
p-eNOS	phosphorylated endothelial NO-synthase
PKG	protein kinase G
p-NGAL	plasma neutrophil gelatinase associated lipocalin
PPARα	Peroxisome proliferator-activated receptor α
PPARγ	Peroxisome proliferator-activated receptor γ
RCT	randomized controlled trial
RGF	reduced graft function
ROS	reactive oxygen species
RT-PCR	real time polymerase chain reaction
SIRT1	Sirtuin-1
SOD	superoxide dismutase
SOT	solid organ transplantation
STAT3	Signal transducer and activator of transcription 3
TBARS	Thiobarbituric acid reactive substances
TLRs	toll-like receptors
TLR4	toll-like receptor 4
TNF-α	tumor necrosis factor α
TnT	cardiac troponin T
Treg	regulatory T-lymphocytes
UW	University of Wisconsin
VEGF-B	vascular endothelial growth factor B
VSMC	vascular smooth muscle cell
WBC	white blood cell
XO	xanthine oxidase
